# Supplementary figures and images for: Tgif1-deficiency impairs cytoskeletal architecture in osteoblasts by activating PAK3 signaling
Source: eLife. 2024 Apr 25;13:RP94265. doi: 10.7554/eLife.94265 (PMC11045221; doi:10.7554/eLife.94265)

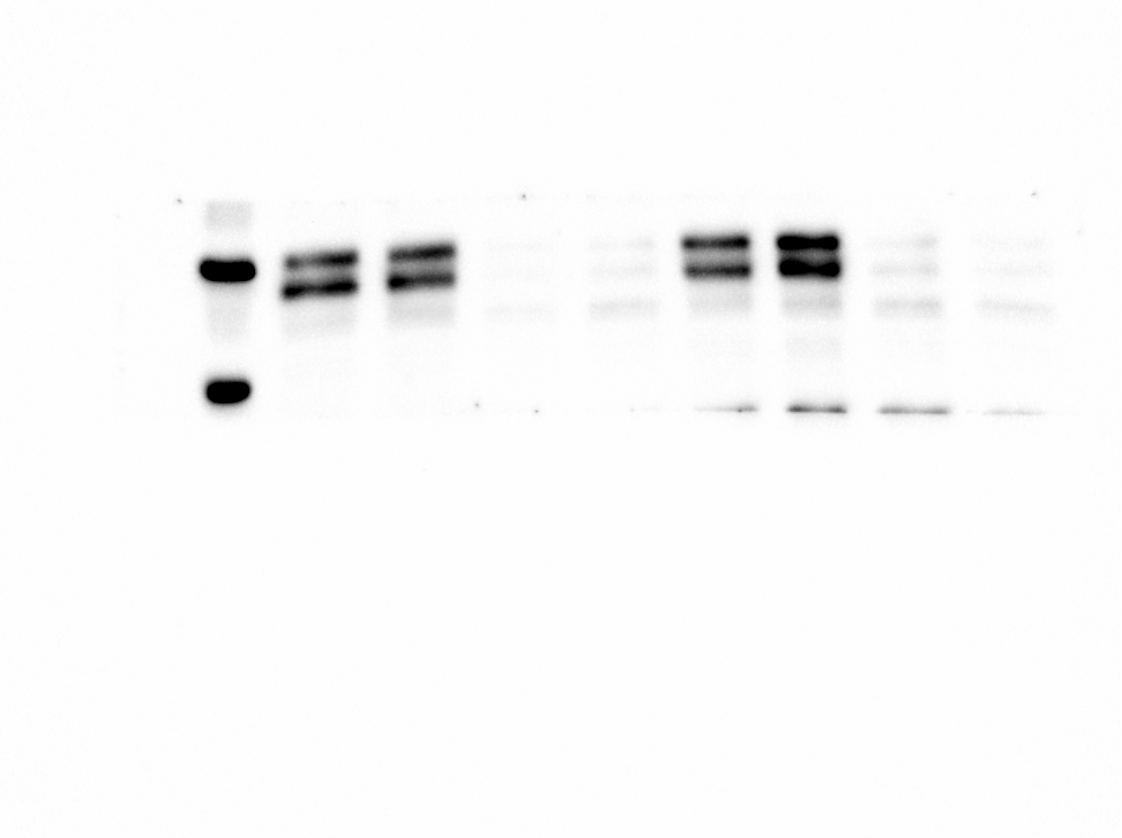

Supplement: Figure 2—figure supplement 1—source data 1. [file elife-94265-fig2-figsupp1-data1.zip › Figure 2-figure supplement 1-source data 1/Figure 2 supplement 1-source data 1_1.tif]

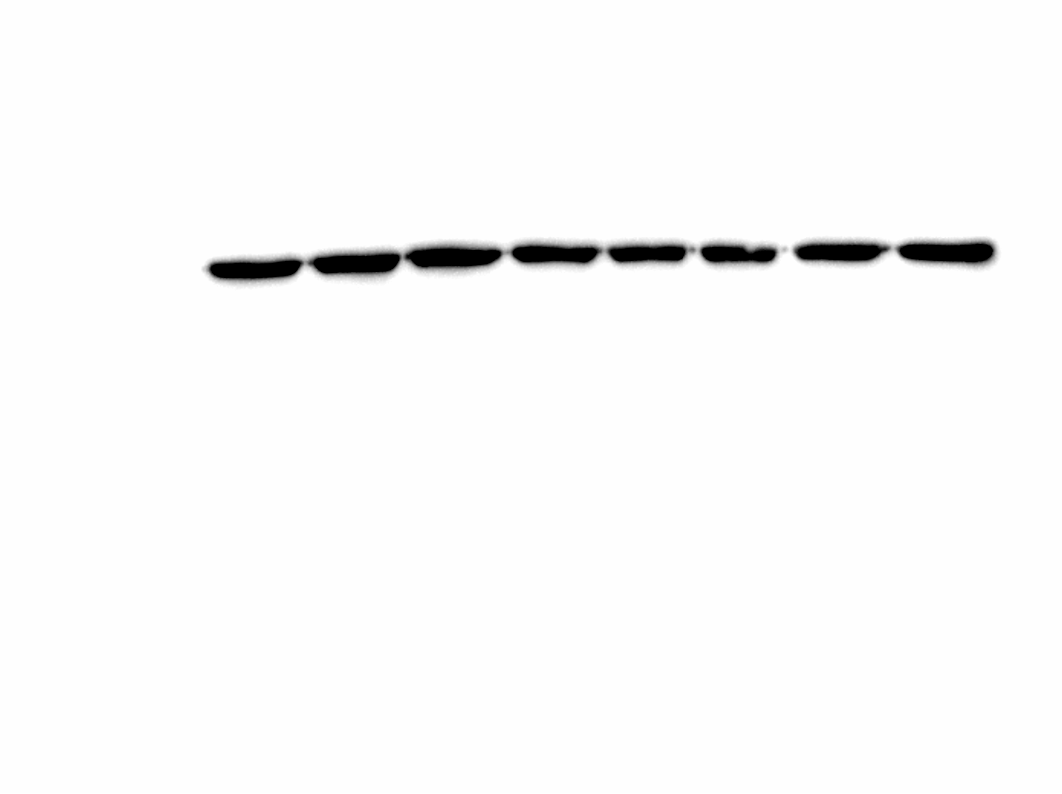

Supplement: Figure 2—figure supplement 1—source data 1. [file elife-94265-fig2-figsupp1-data1.zip › Figure 2-figure supplement 1-source data 1/Figure 2 supplement 1-source data 1_2.tif]

**B**

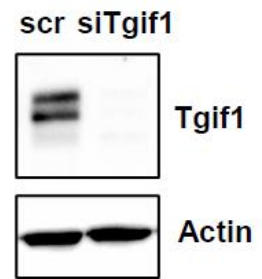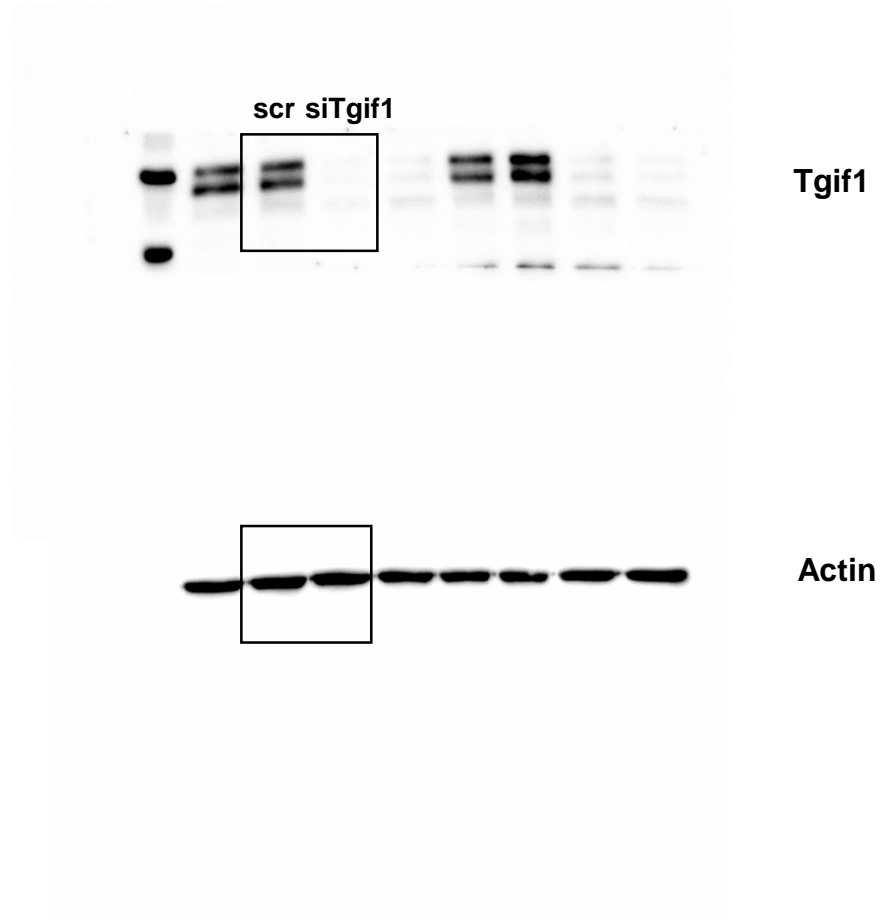

Figure 2- supplement 1

Supplement: Figure 2—figure supplement 1—source data 2. [file elife-94265-fig2-figsupp1-data2.zip › Figure 2-figure supplement 1-source data 2/Figure 2- supplement 1-data source 2.pdf]

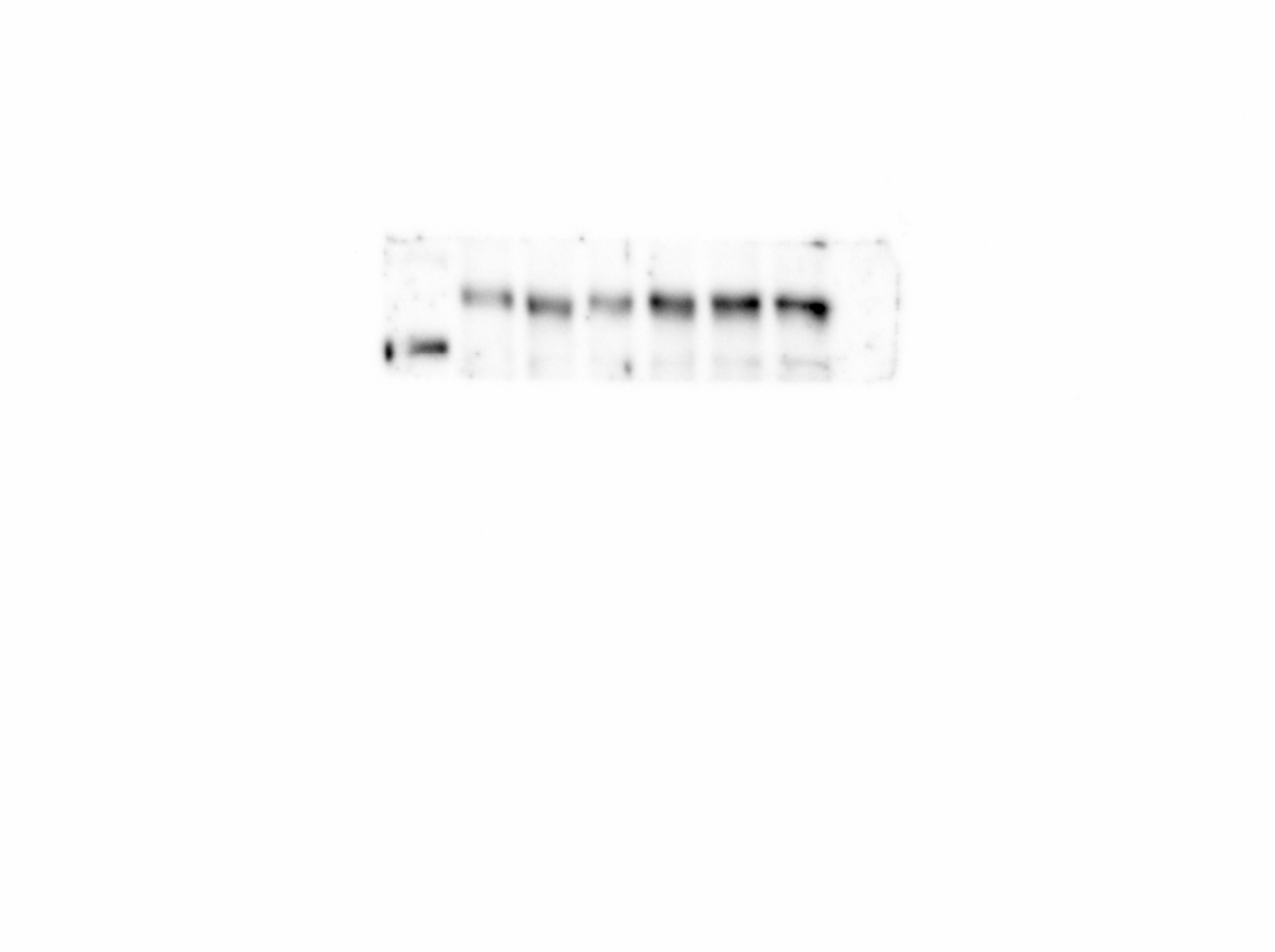

Supplement: Figure 3—source data 2. [file elife-94265-fig3-data2.zip › Figure 3-source data 2/Figure 3-source data 2_1.tif]

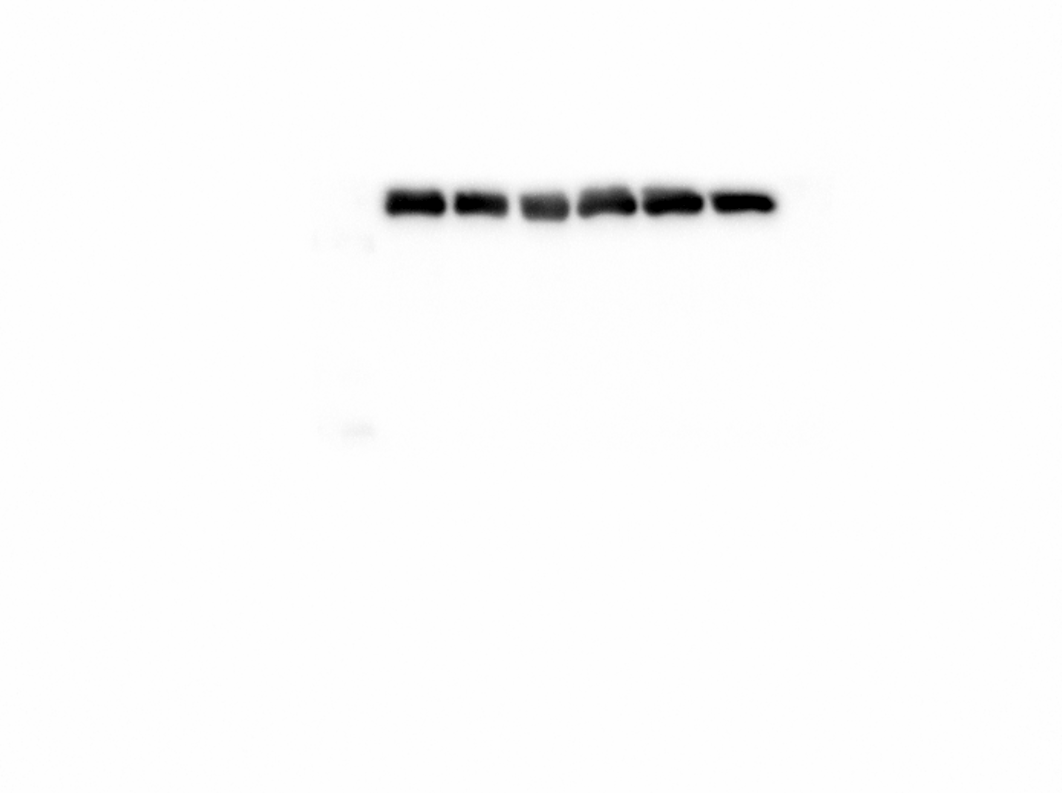

Supplement: Figure 3—source data 2. [file elife-94265-fig3-data2.zip › Figure 3-source data 2/Figure 3-source data 2_2.tif]

**B**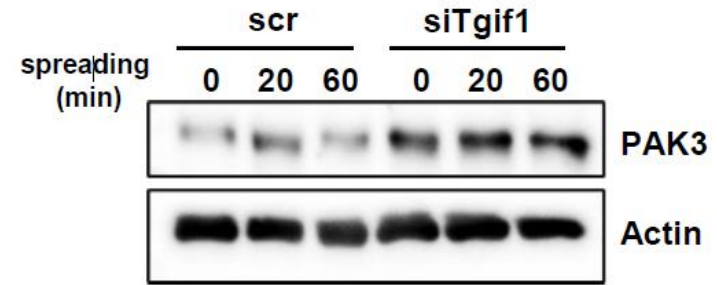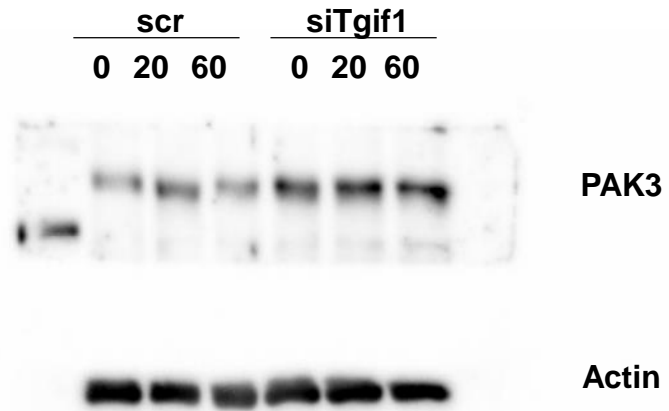

Figure 3B

Supplement: Figure 3—source data 3. [file elife-94265-fig3-data3.zip › Figure 3-source data 3/Figure 3B-source data 3.pdf]

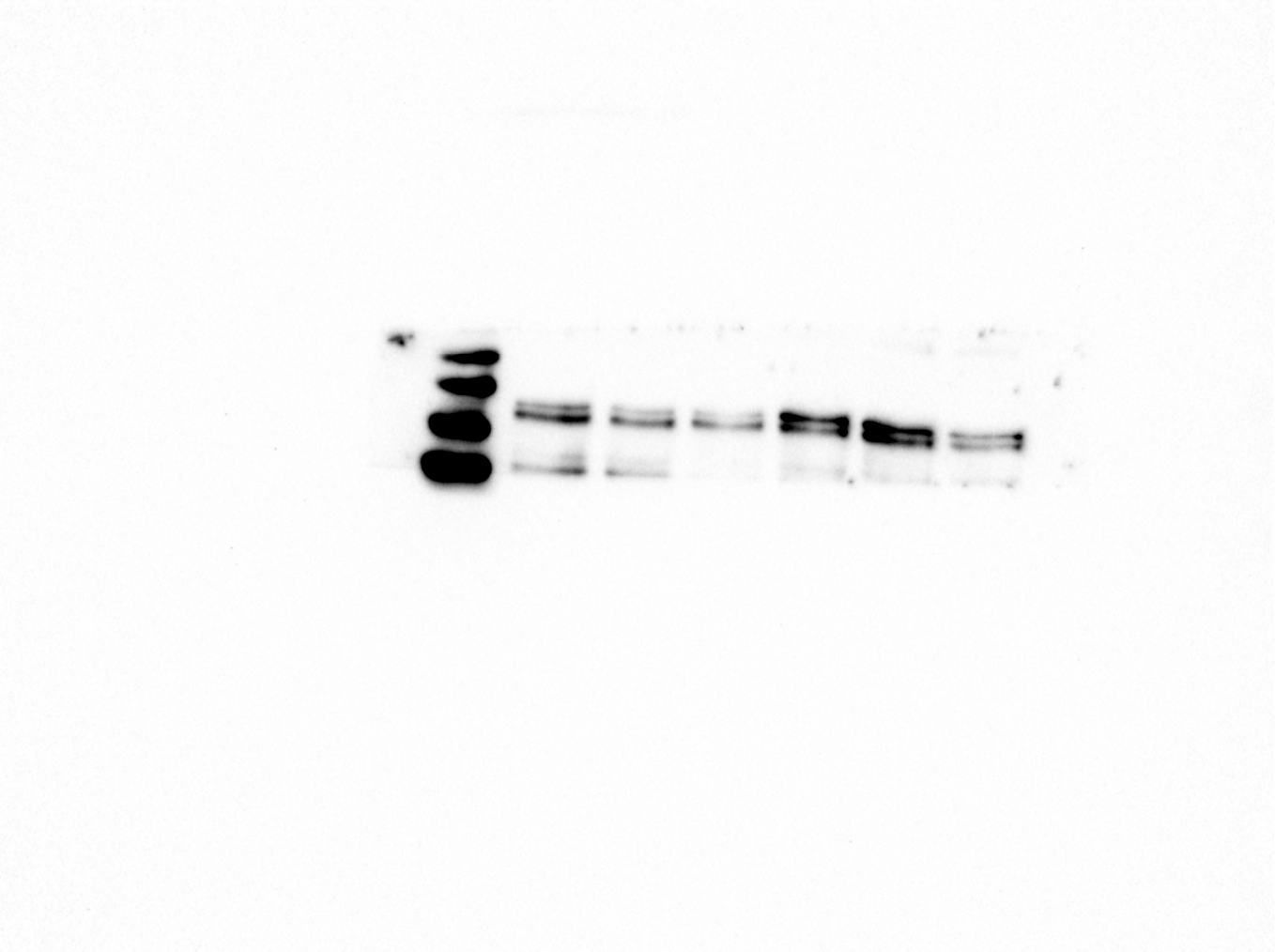

Supplement: Figure 3—figure supplement 1—source data 1. [file elife-94265-fig3-figsupp1-data1.zip › Figure 3-figure supplement 1-source data 1/Figure 3 supplement 1-source data 1_1.tif]

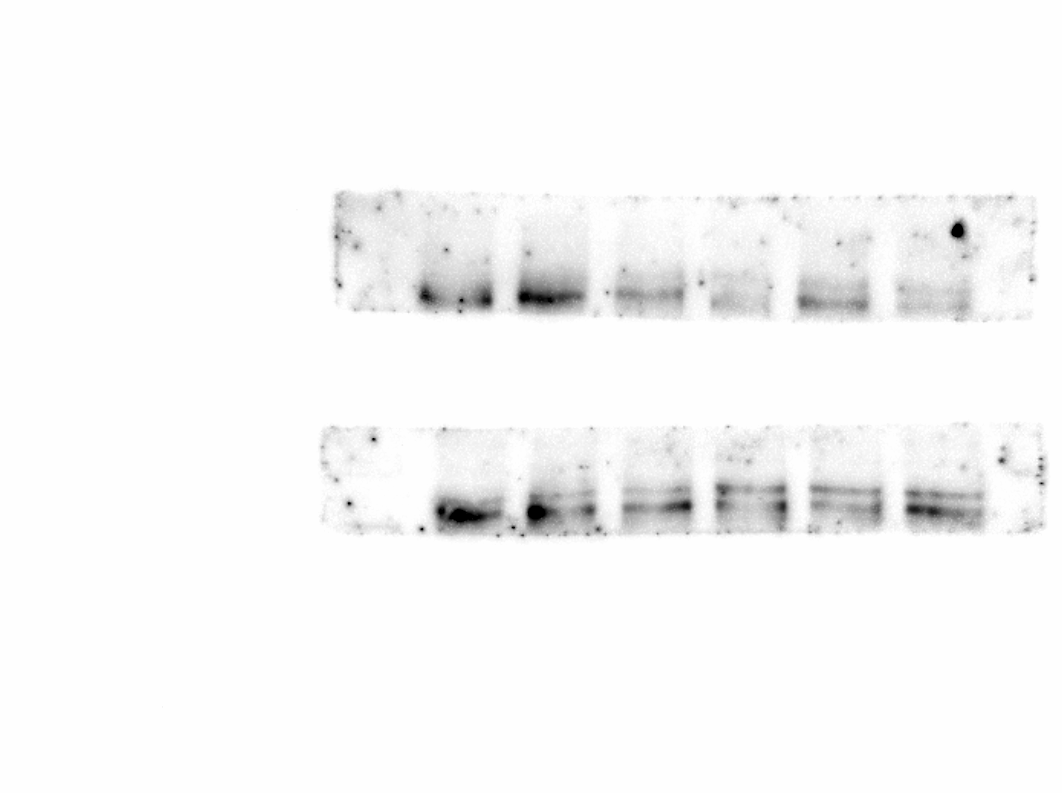

Supplement: Figure 3—figure supplement 1—source data 1. [file elife-94265-fig3-figsupp1-data1.zip › Figure 3-figure supplement 1-source data 1/Figure 3 supplement 1-source data 1_10.tif]

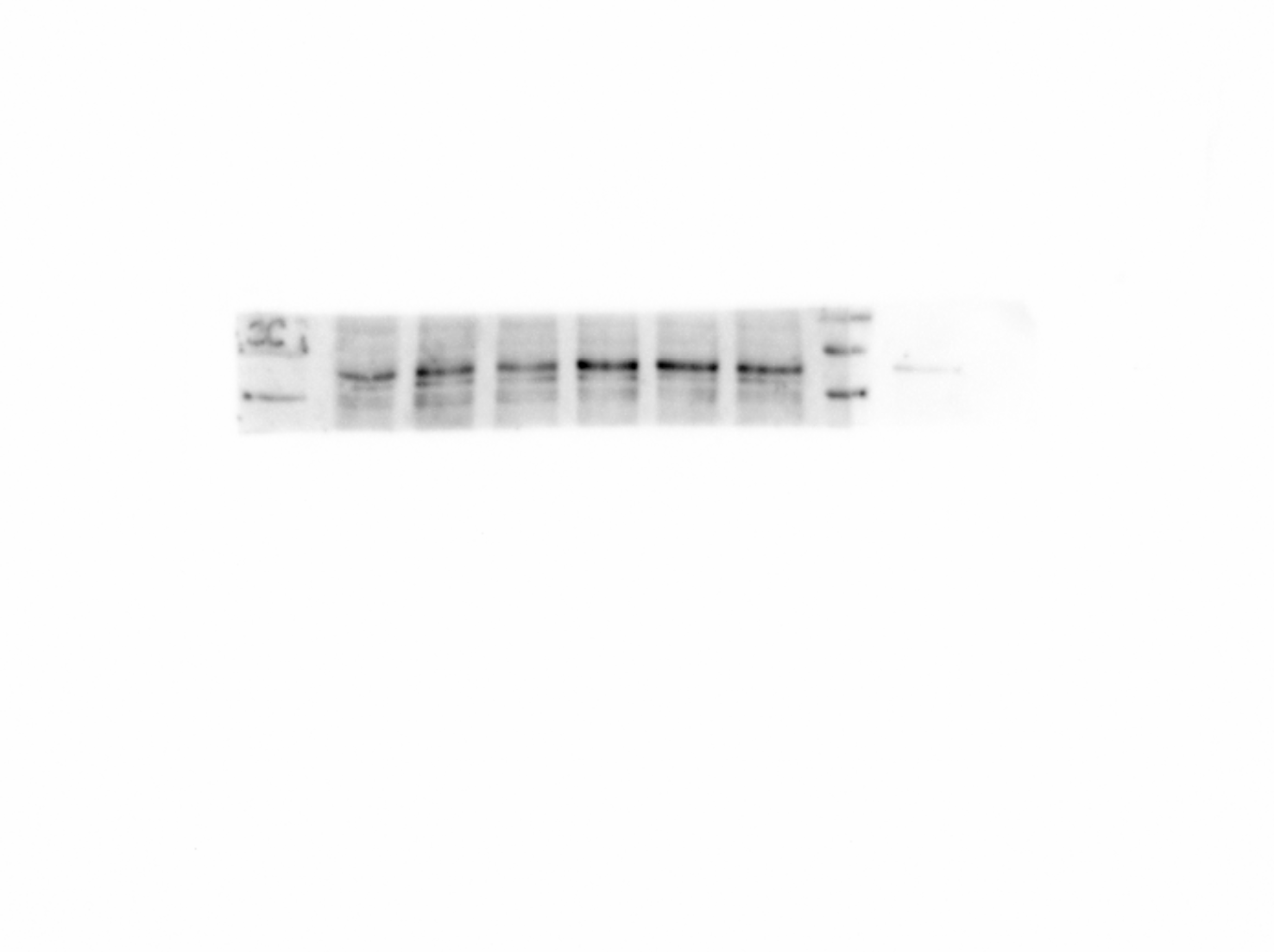

Supplement: Figure 3—figure supplement 1—source data 1. [file elife-94265-fig3-figsupp1-data1.zip › Figure 3-figure supplement 1-source data 1/Figure 3 supplement 1-source data 1_11.tif]

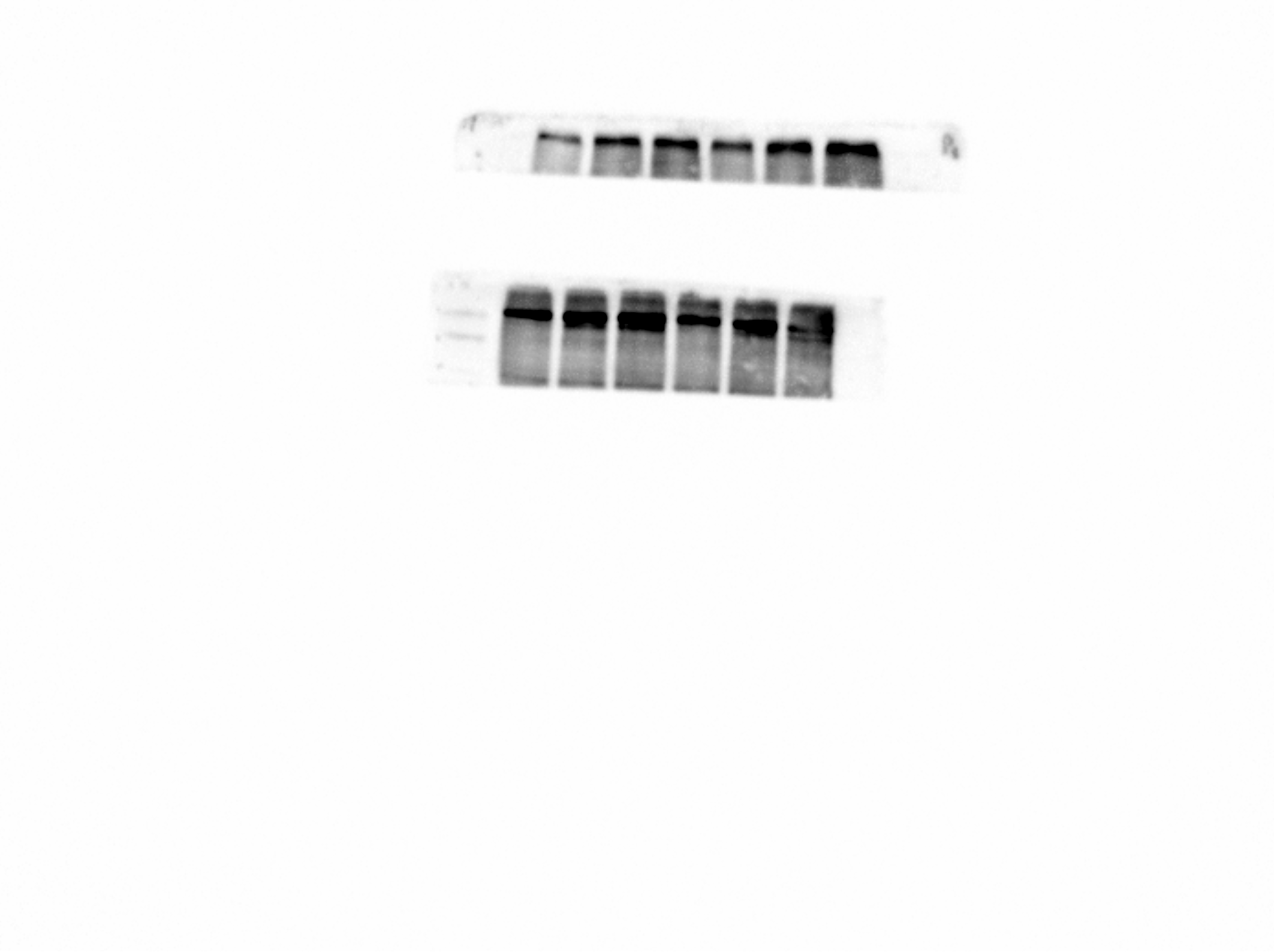

Supplement: Figure 3—figure supplement 1—source data 1. [file elife-94265-fig3-figsupp1-data1.zip › Figure 3-figure supplement 1-source data 1/Figure 3 supplement 1-source data 1_12.tif]

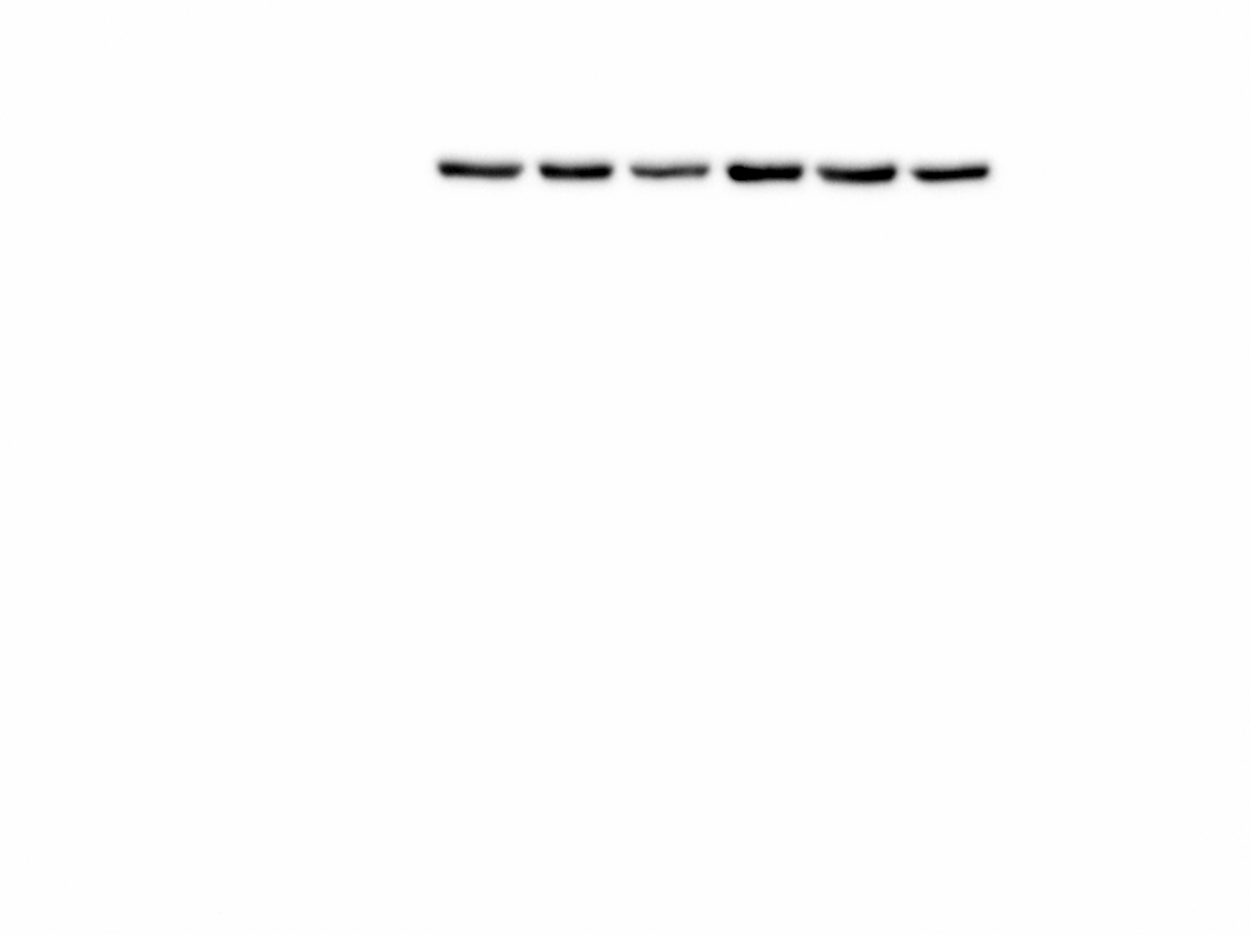

Supplement: Figure 3—figure supplement 1—source data 1. [file elife-94265-fig3-figsupp1-data1.zip › Figure 3-figure supplement 1-source data 1/Figure 3 supplement 1-source data 1_13.tif]

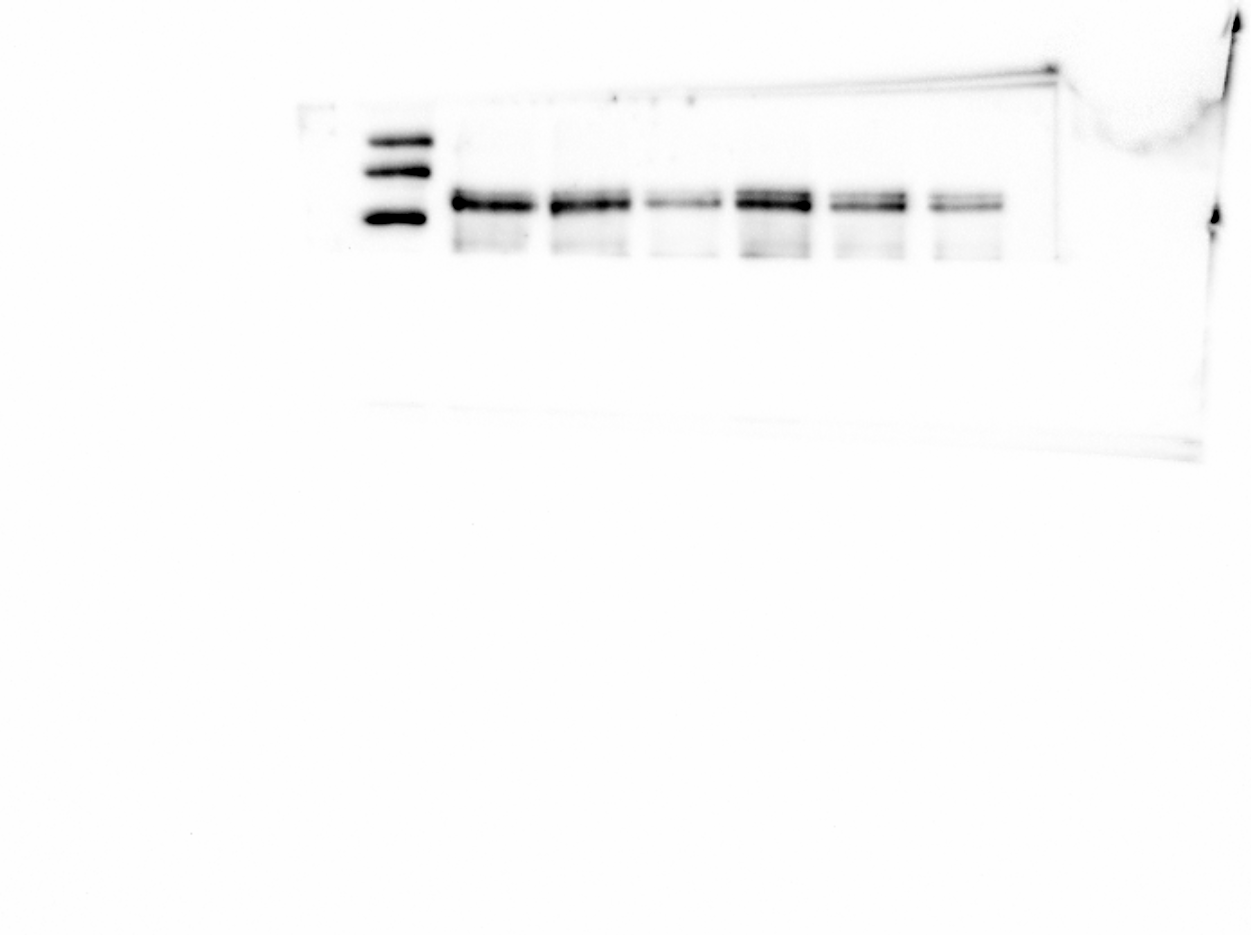

Supplement: Figure 3—figure supplement 1—source data 1. [file elife-94265-fig3-figsupp1-data1.zip › Figure 3-figure supplement 1-source data 1/Figure 3 supplement 1-source data 1_2.tif]

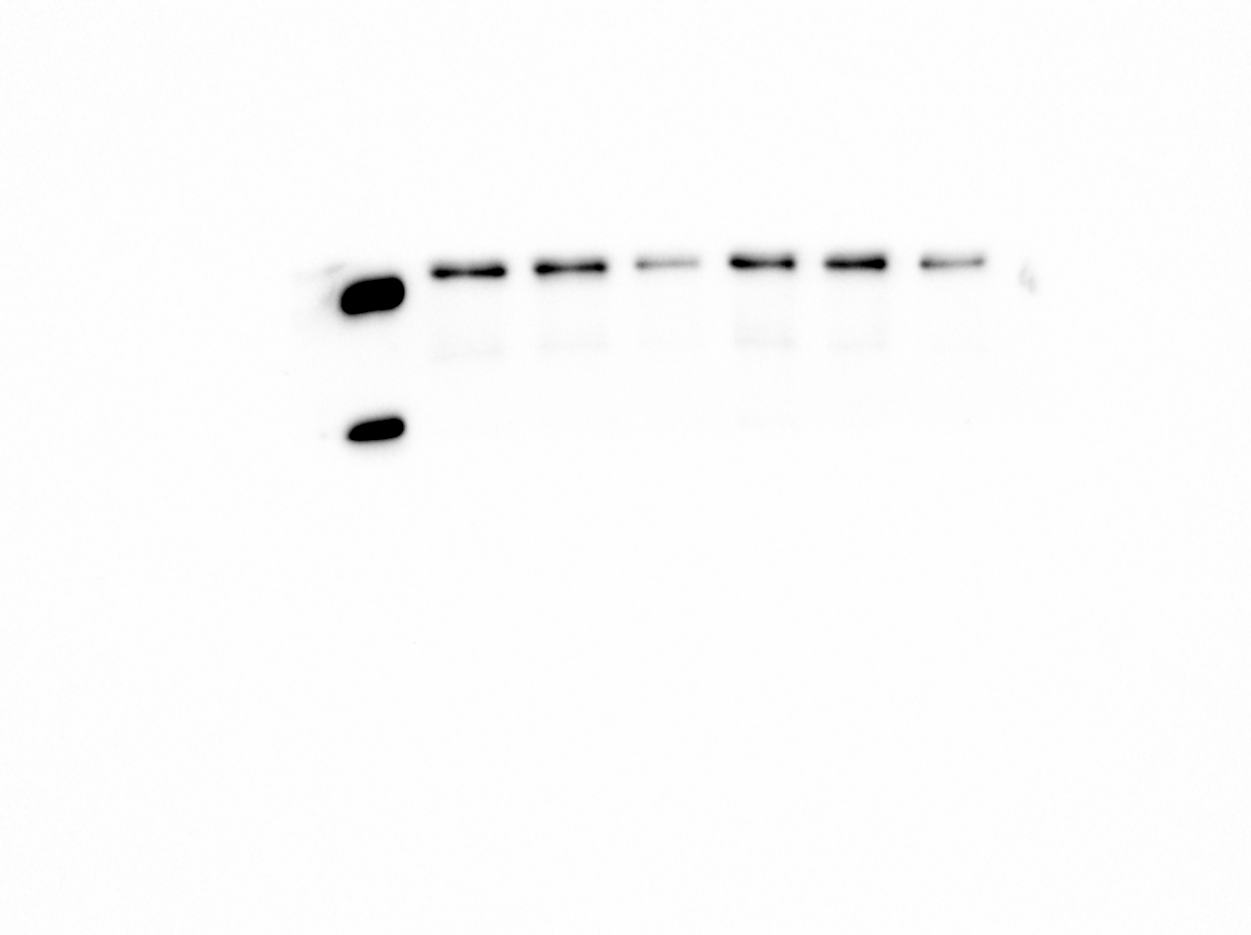

Supplement: Figure 3—figure supplement 1—source data 1. [file elife-94265-fig3-figsupp1-data1.zip › Figure 3-figure supplement 1-source data 1/Figure 3 supplement 1-source data 1_3.tif]

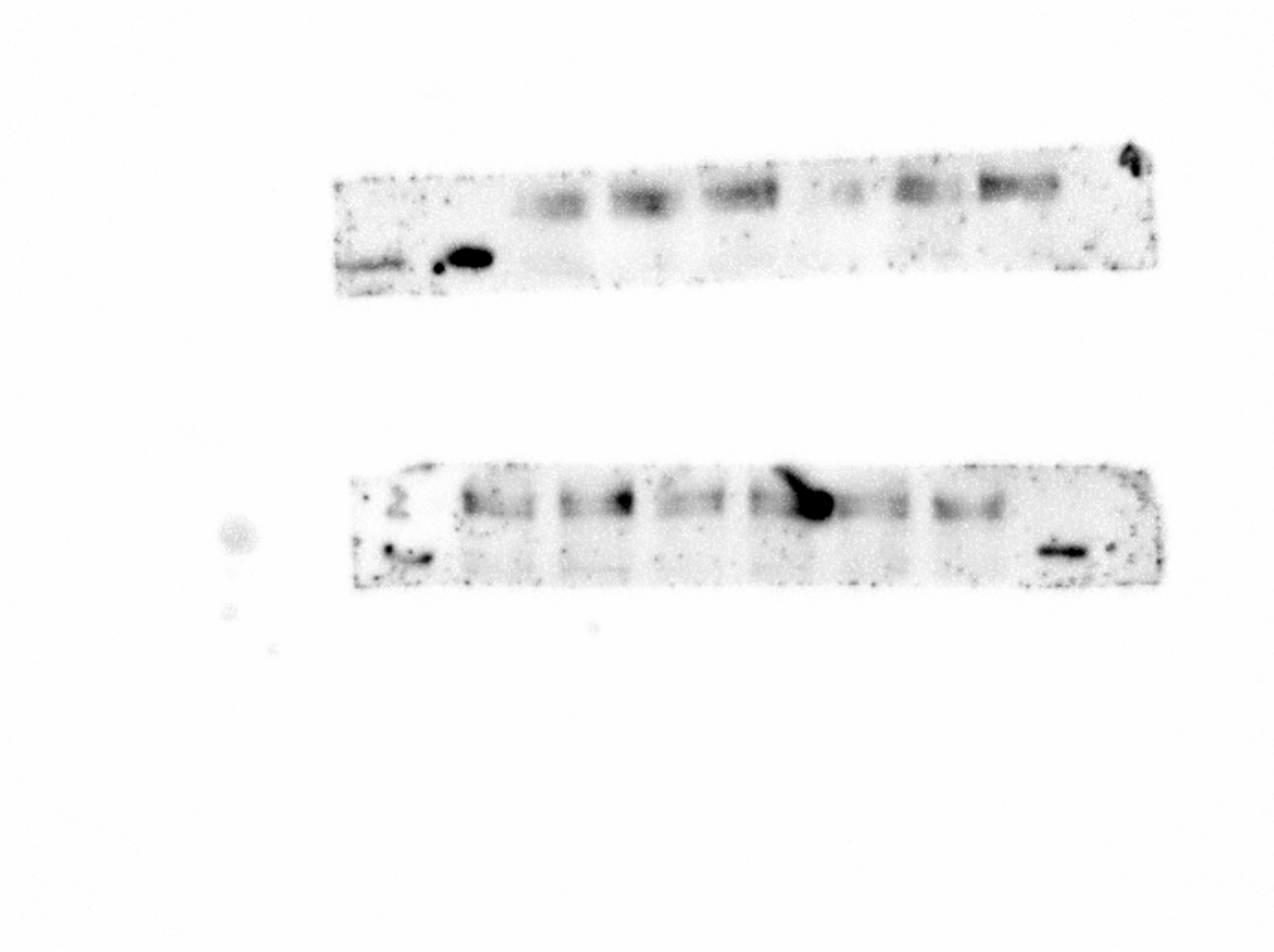

Supplement: Figure 3—figure supplement 1—source data 1. [file elife-94265-fig3-figsupp1-data1.zip › Figure 3-figure supplement 1-source data 1/Figure 3 supplement 1-source data 1_5.tif]

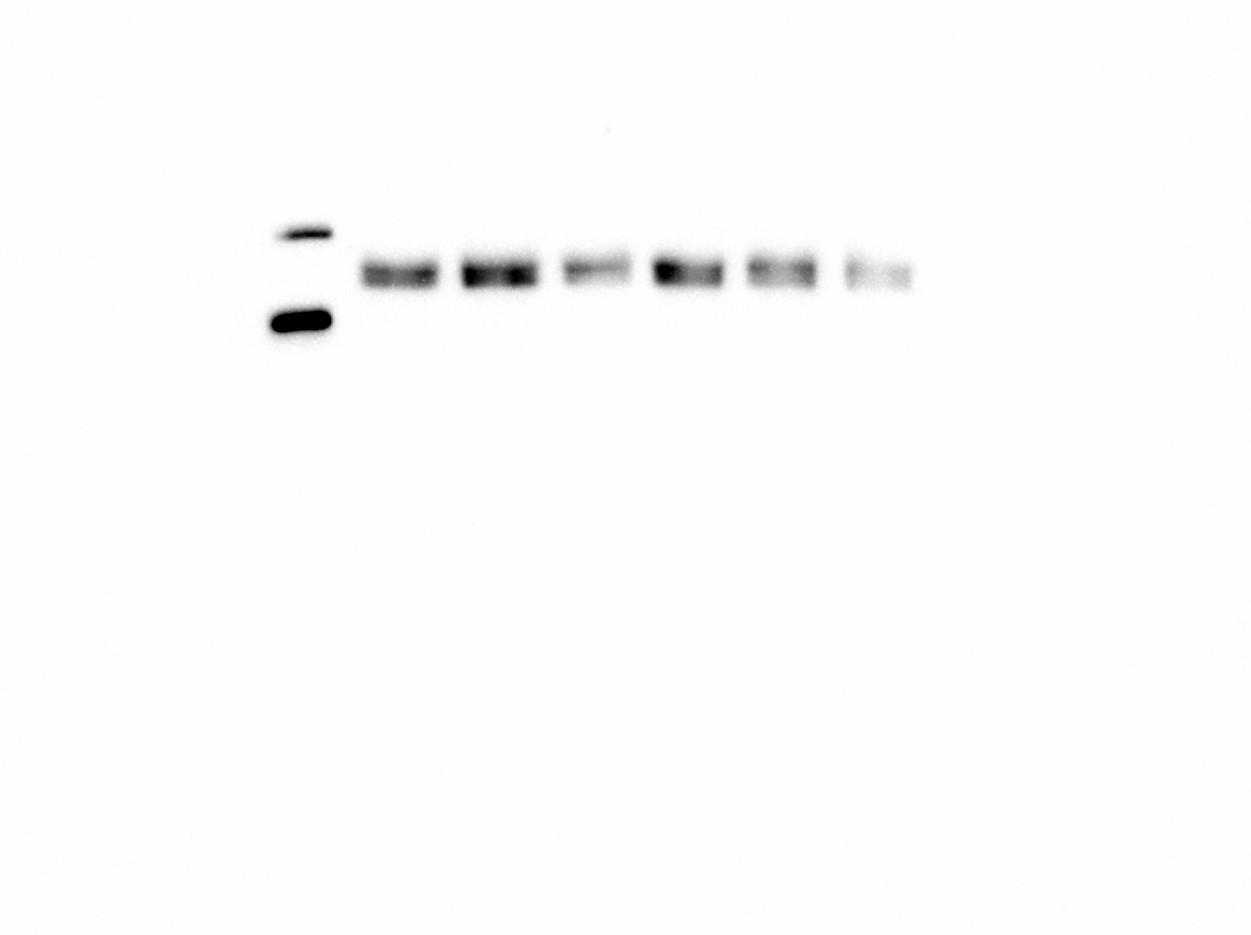

Supplement: Figure 3—figure supplement 1—source data 1. [file elife-94265-fig3-figsupp1-data1.zip › Figure 3-figure supplement 1-source data 1/Figure 3 supplement 1-source data 1_6.tif]

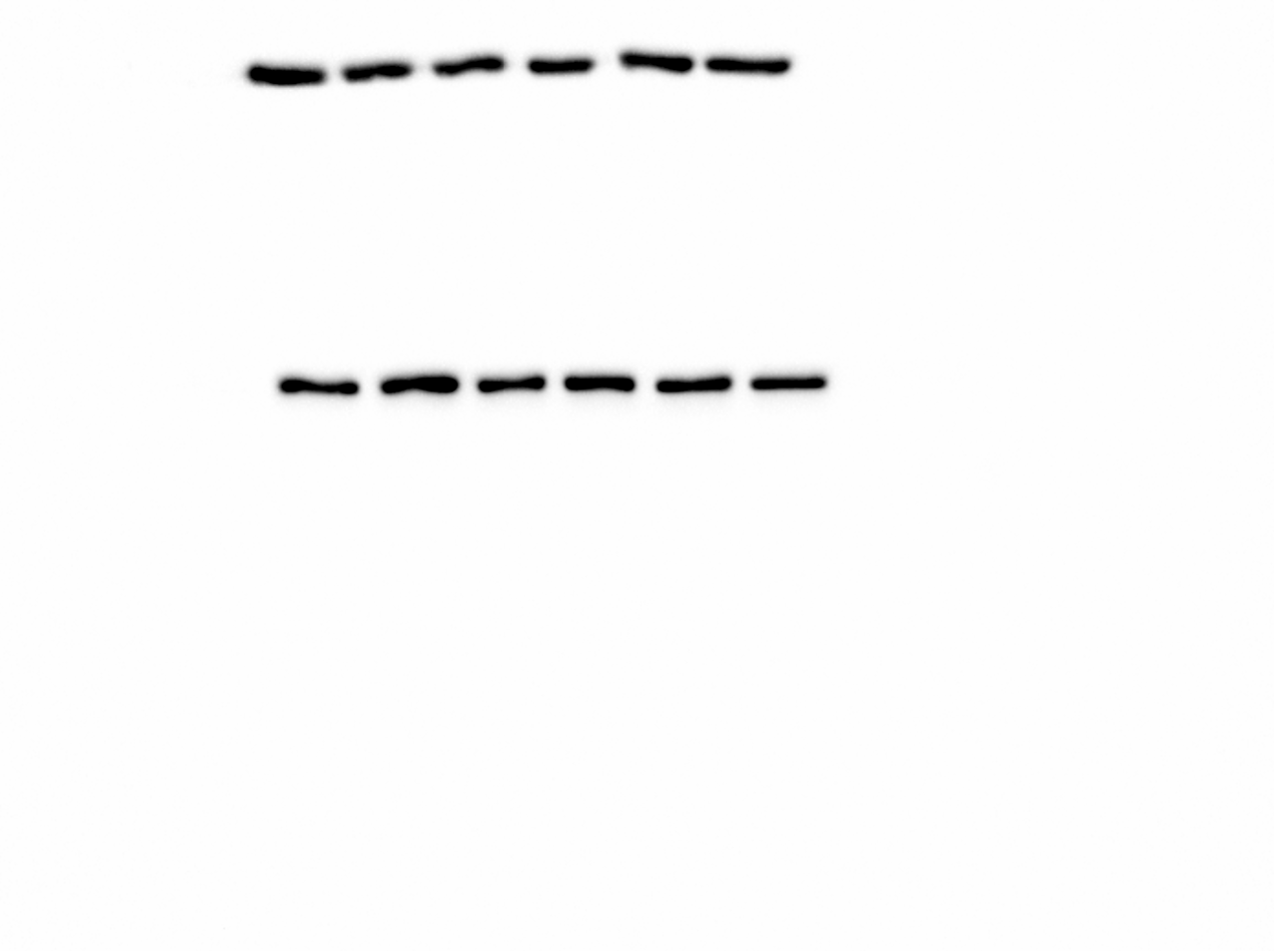

Supplement: Figure 3—figure supplement 1—source data 1. [file elife-94265-fig3-figsupp1-data1.zip › Figure 3-figure supplement 1-source data 1/Figure 3 supplement 1-source data 1_7.tif]

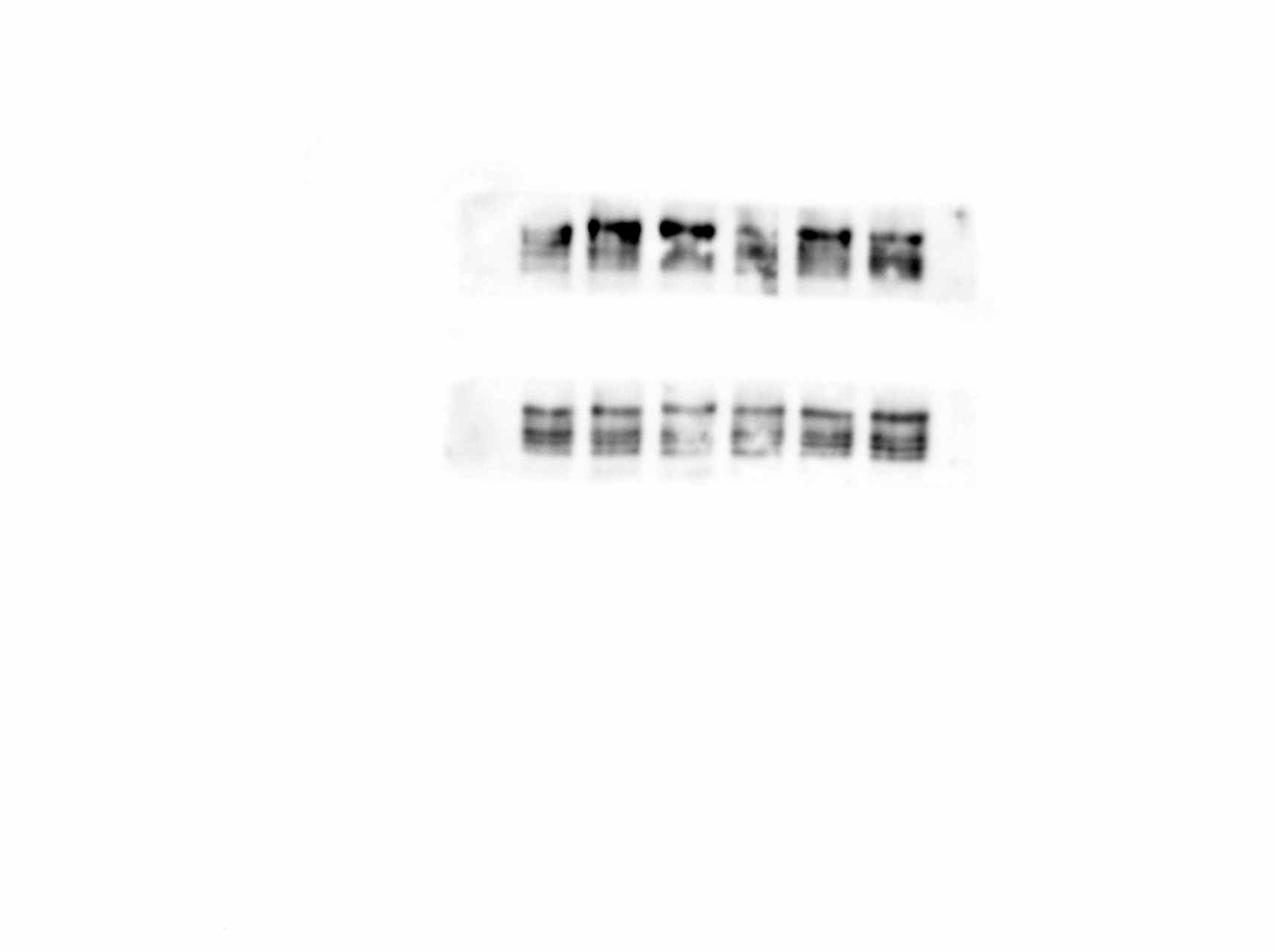

Supplement: Figure 3—figure supplement 1—source data 1. [file elife-94265-fig3-figsupp1-data1.zip › Figure 3-figure supplement 1-source data 1/Figure 3 supplement 1-source data 1_8.tif]

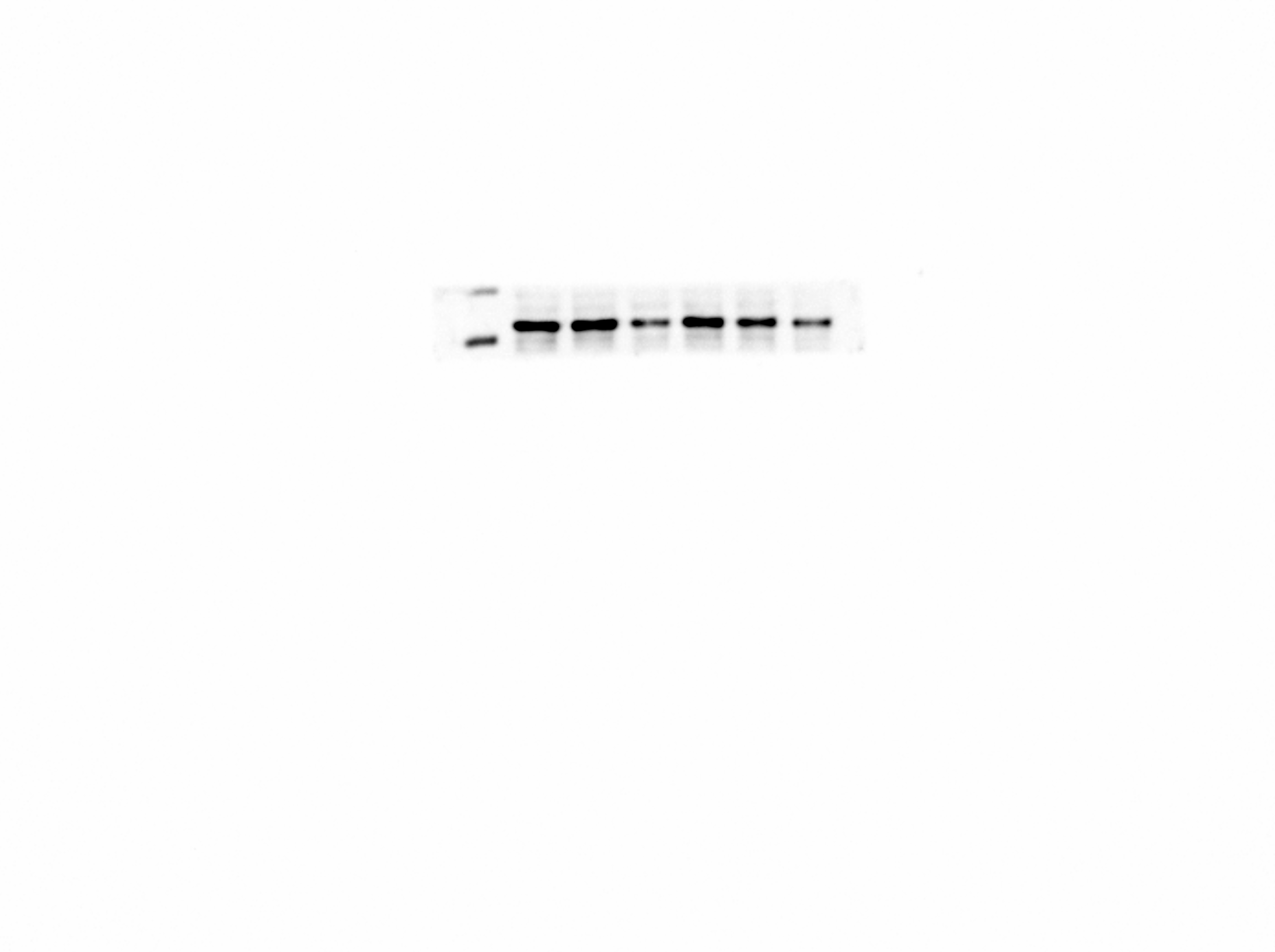

Supplement: Figure 3—figure supplement 1—source data 1. [file elife-94265-fig3-figsupp1-data1.zip › Figure 3-figure supplement 1-source data 1/Figure 3 supplement 1-source data 1_9.tif]

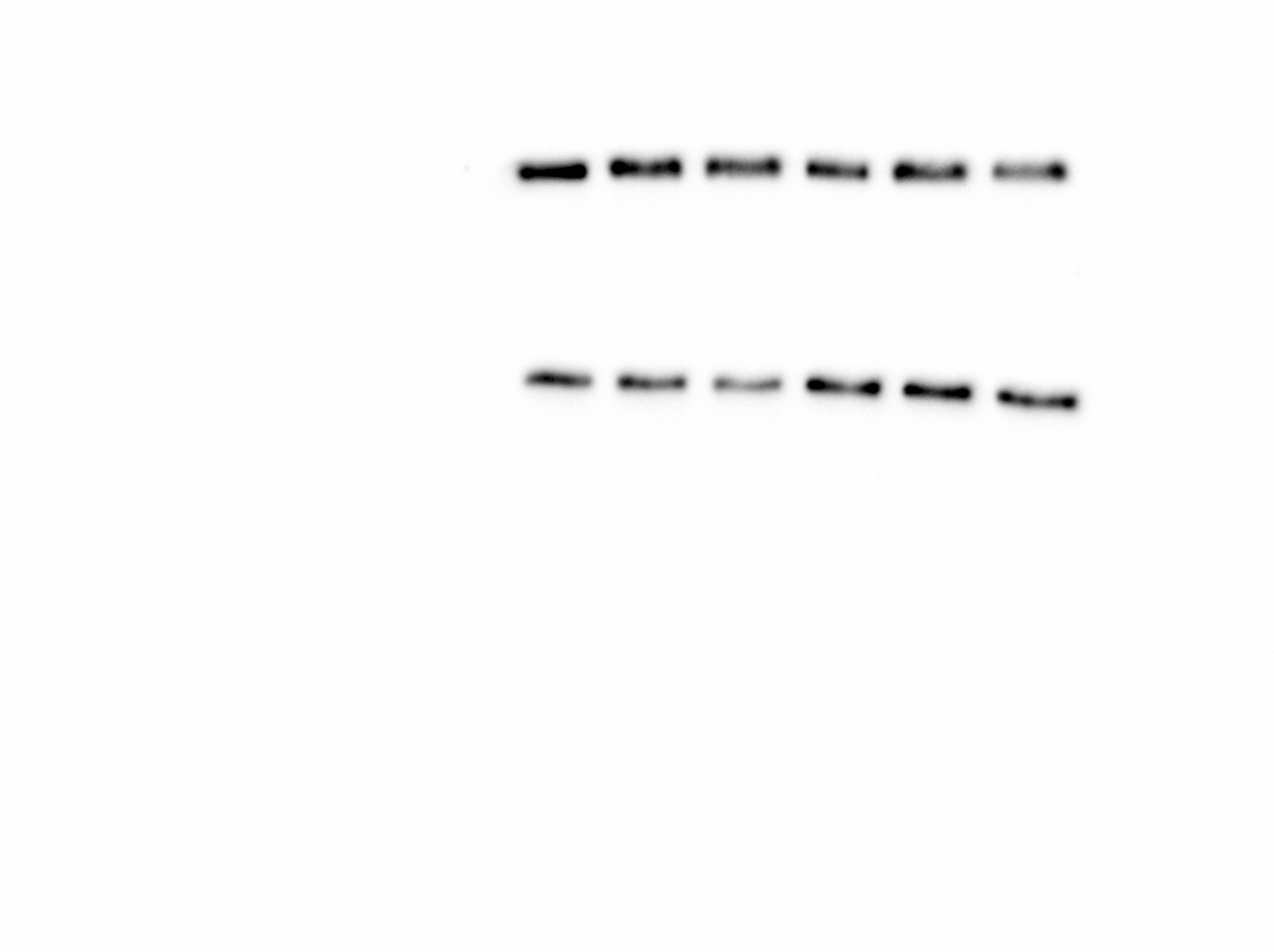

Supplement: Figure 3—figure supplement 1—source data 1. [file elife-94265-fig3-figsupp1-data1.zip › Figure 3-figure supplement 1-source data 1/Figure 3 supplement 1-source data1_4.tif]

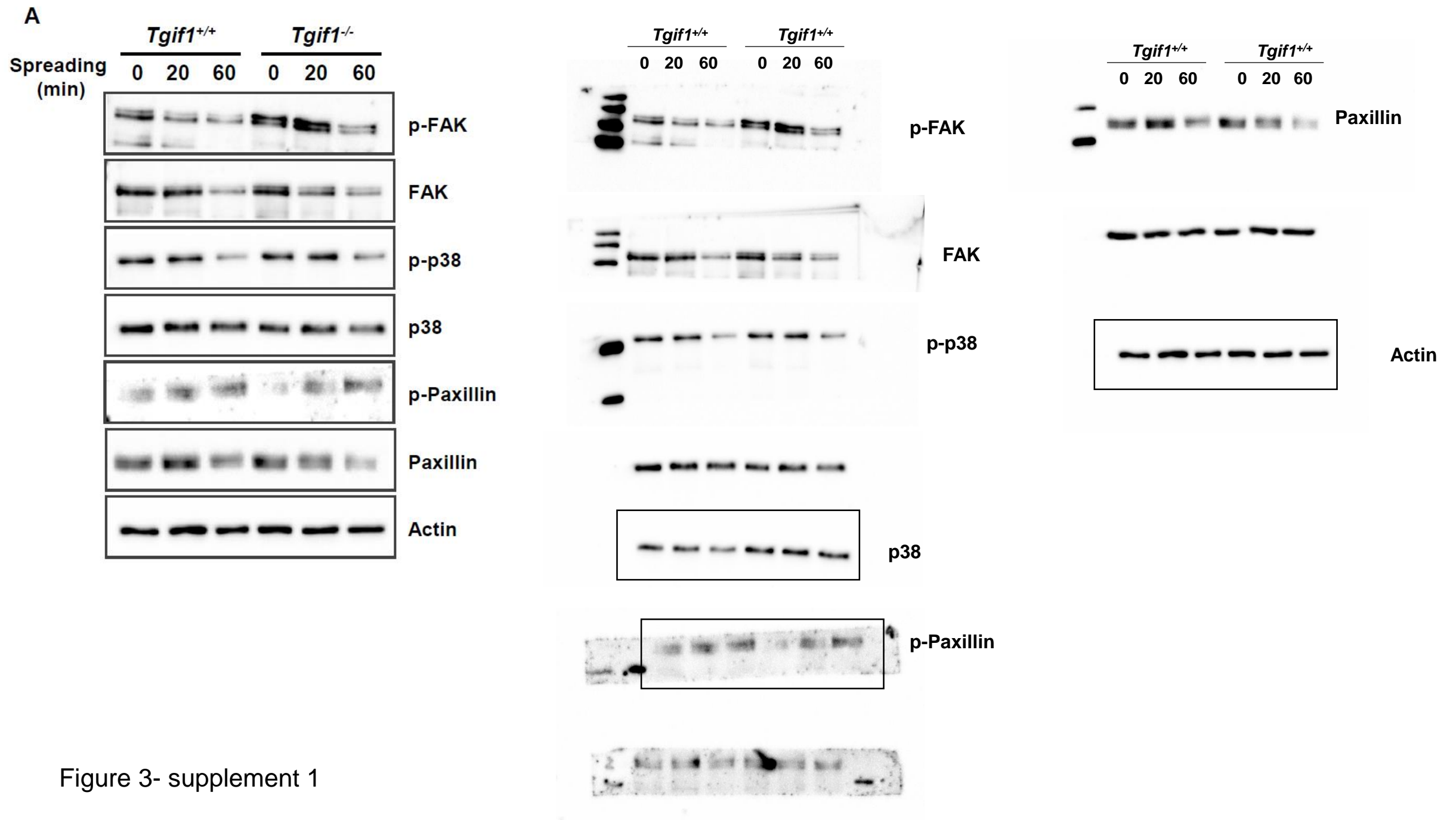

Figure 3- supplement 1

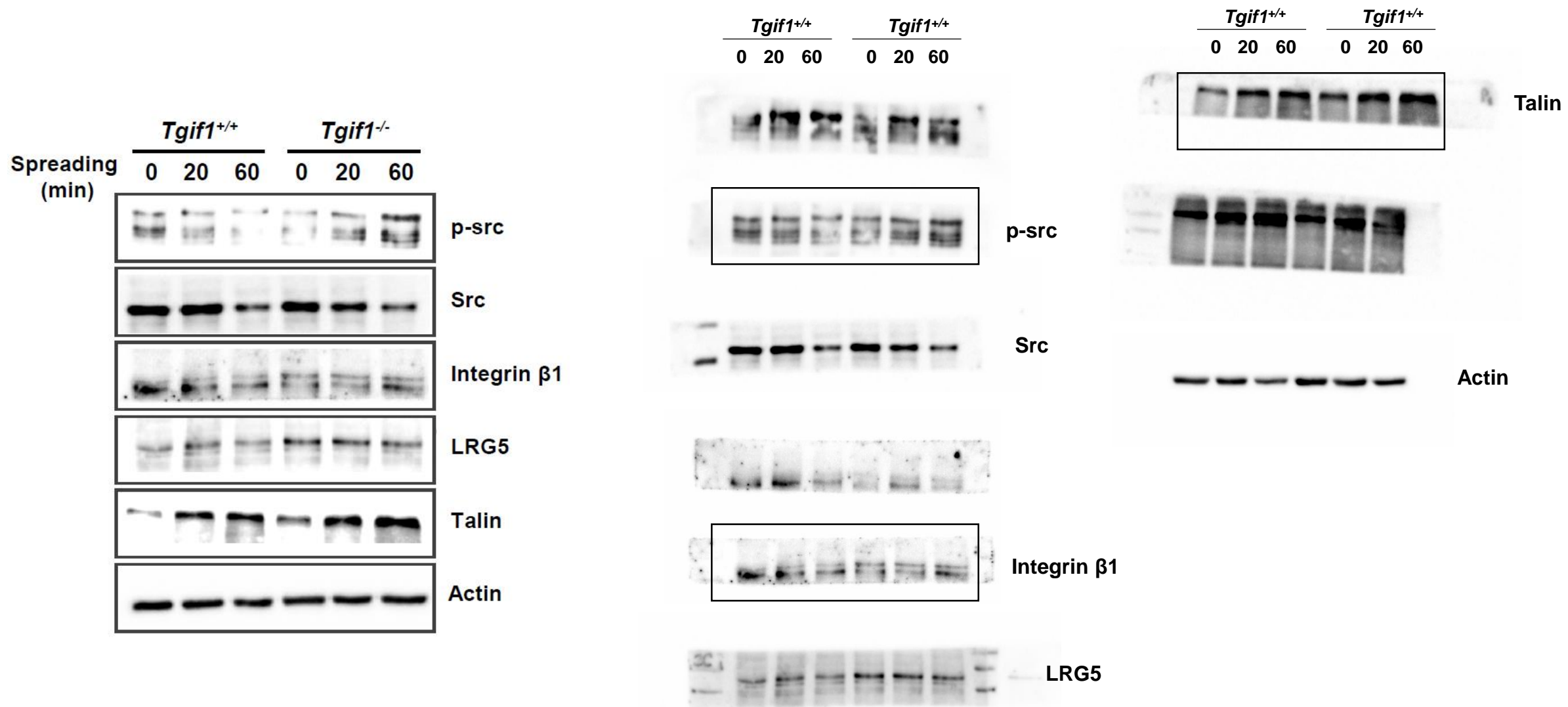

Figure 3- supplement 1

Supplement: Figure 3—figure supplement 1—source data 2. [file elife-94265-fig3-figsupp1-data2.zip › Figure 3-figure supplement 1-source data 2/Figure 3- supplement 1-source data 2.pdf]

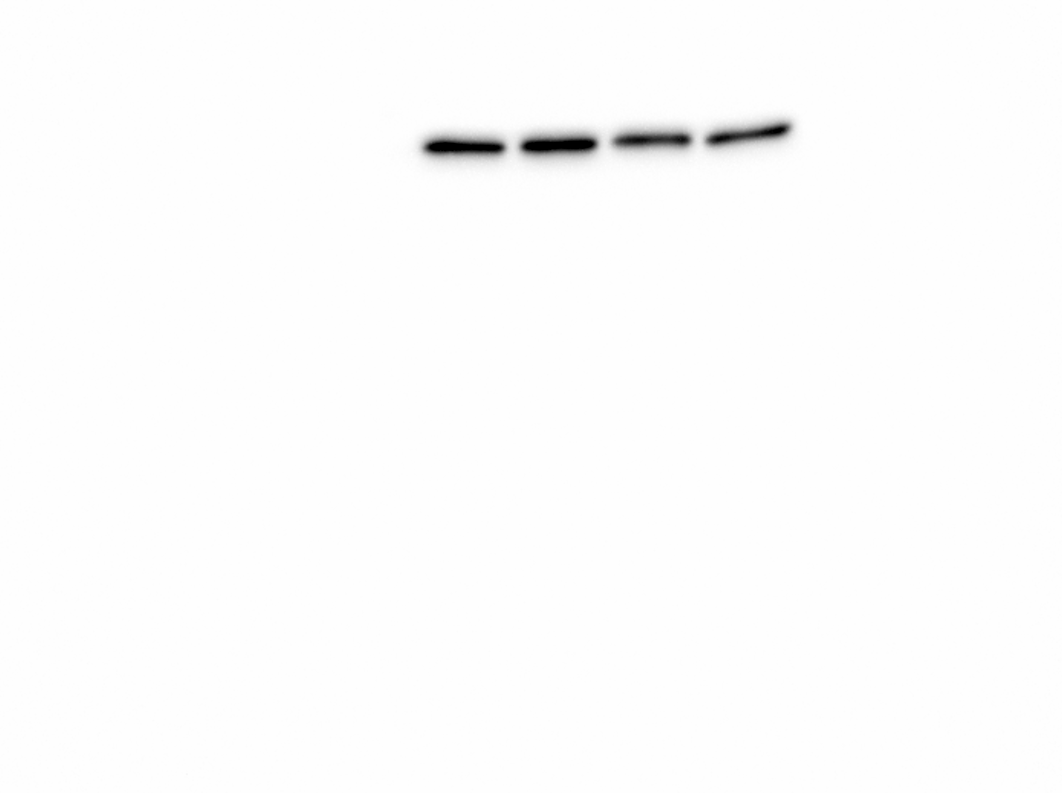

Supplement: Figure 3—figure supplement 3—source data 1. [file elife-94265-fig3-figsupp3-data1.zip › Figure 3-figure supplement 3-source data 1/Figure 3 supplement 3-source data 1_3.tif]

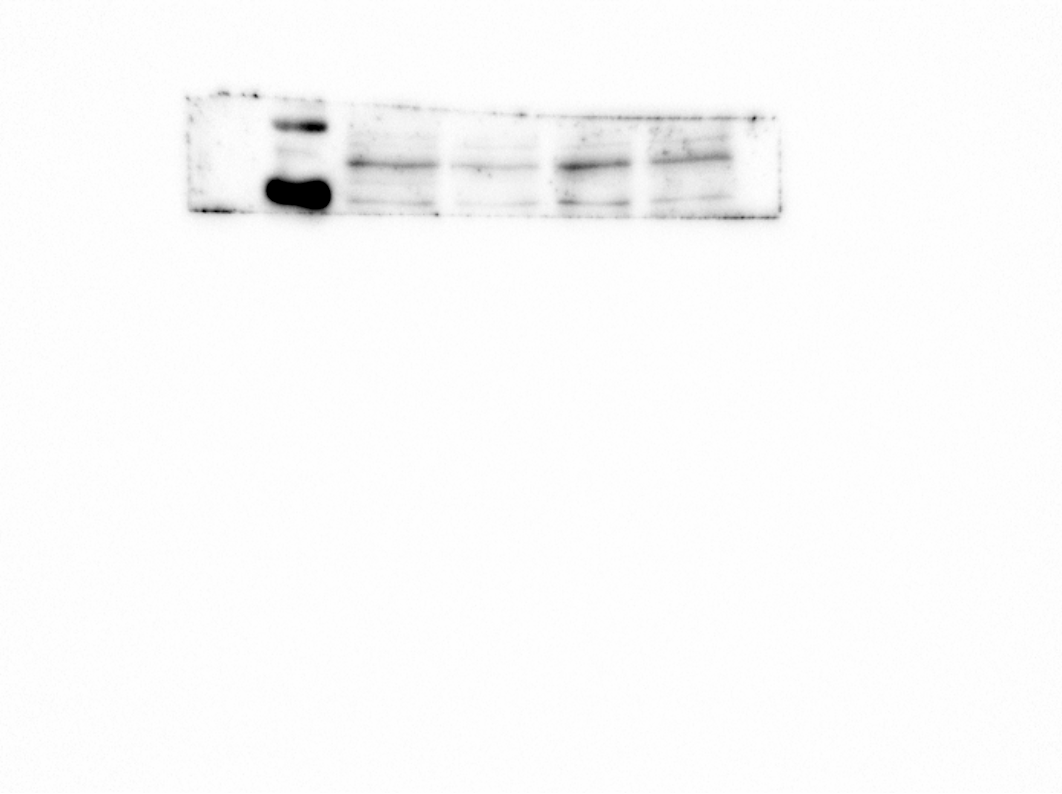

Supplement: Figure 3—figure supplement 3—source data 1. [file elife-94265-fig3-figsupp3-data1.zip › Figure 3-figure supplement 3-source data 1/Figure 3 supplement 3-source data 1_1.tif]

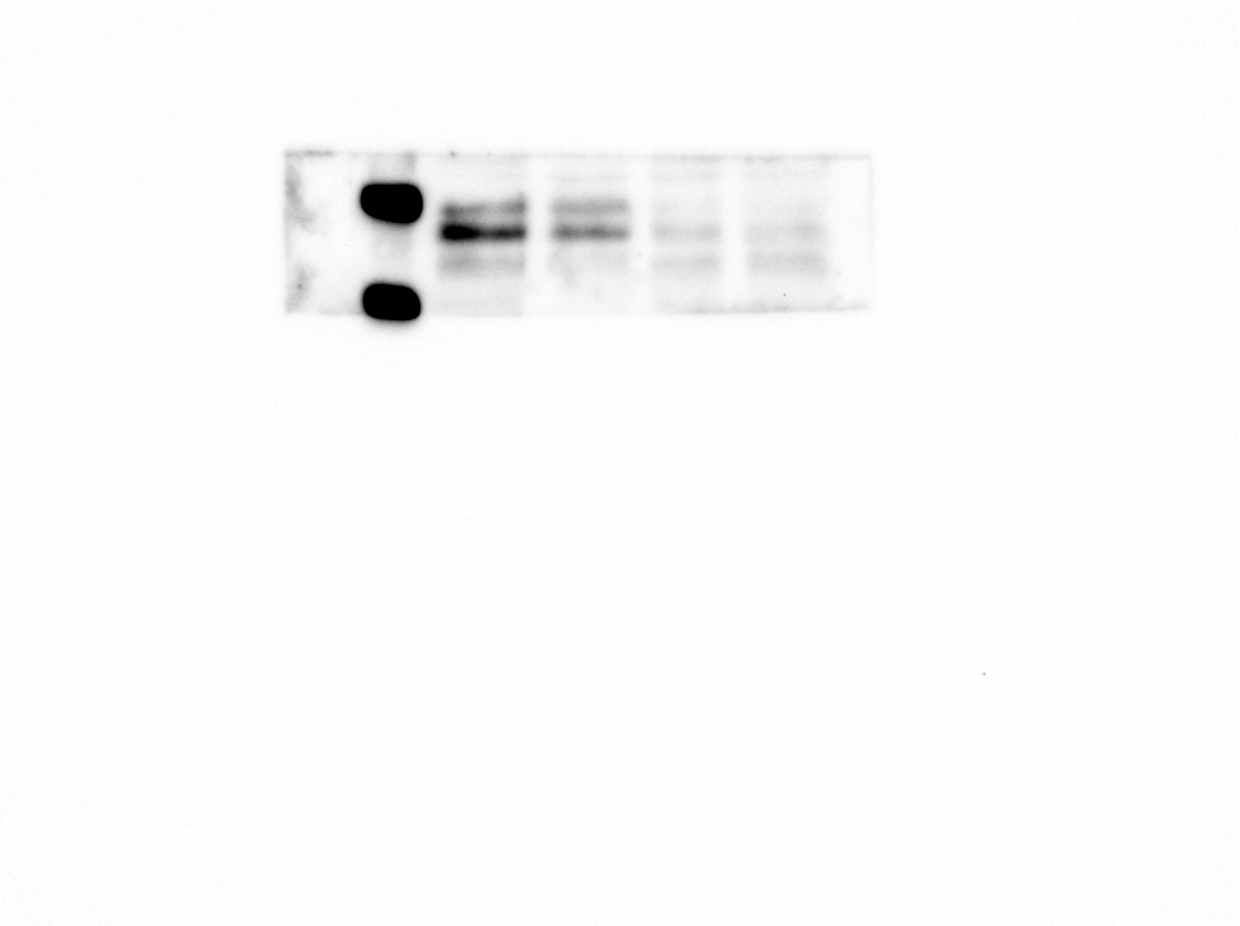

Supplement: Figure 3—figure supplement 3—source data 1. [file elife-94265-fig3-figsupp3-data1.zip › Figure 3-figure supplement 3-source data 1/Figure 3 supplement 3-source data 1_2.tif]

**B**

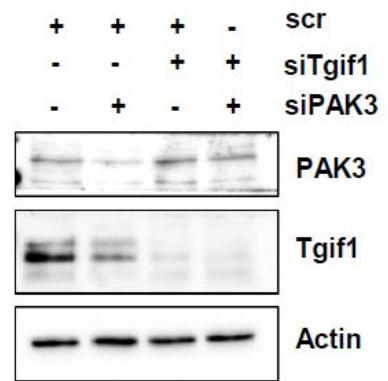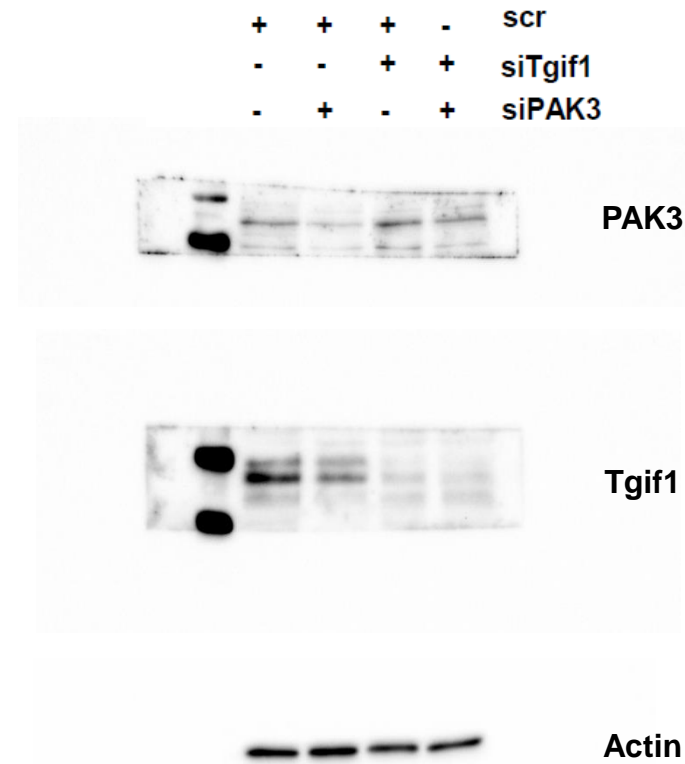

Figure 3- supplement 3

Supplement: Figure 3—figure supplement 3—source data 2. [file elife-94265-fig3-figsupp3-data2.zip › Figure 3-figure supplement 3-source data 2/Figure 3- supplement 3-source data 2.pdf]

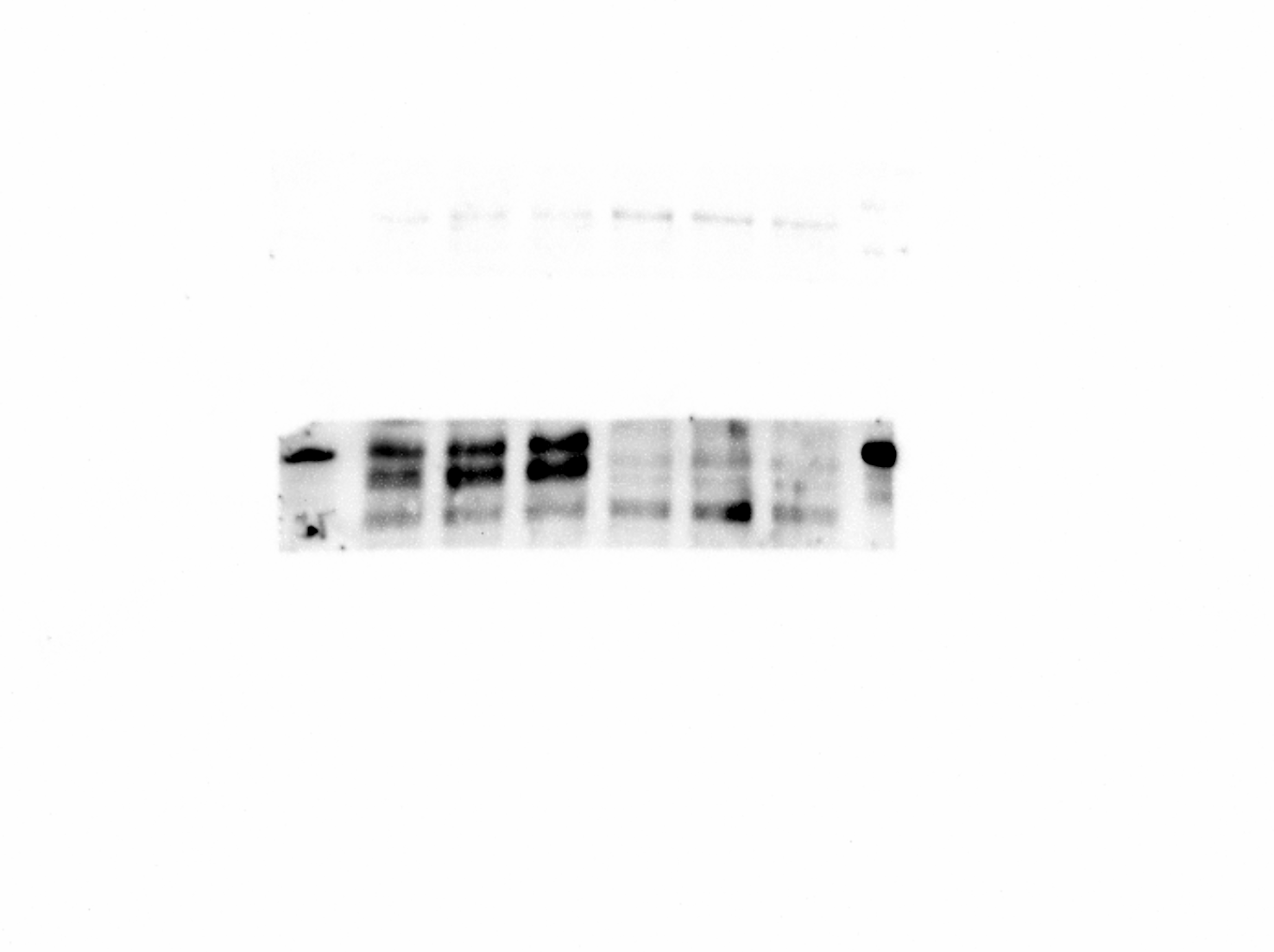

Supplement: Figure 4—source data 2. [file elife-94265-fig4-data2.zip › Figure 4-source data 2/Figure 4 -source data 2_1.tif]

**B**

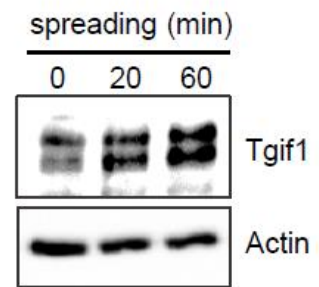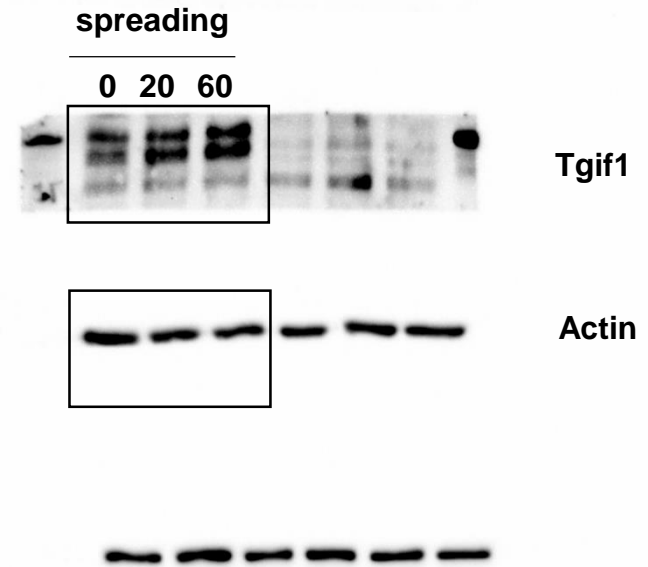

Figure 4B

Supplement: Figure 4—source data 3. [file elife-94265-fig4-data3.zip › Figure 4-source data 3/Figure 4-source data 3.pdf]

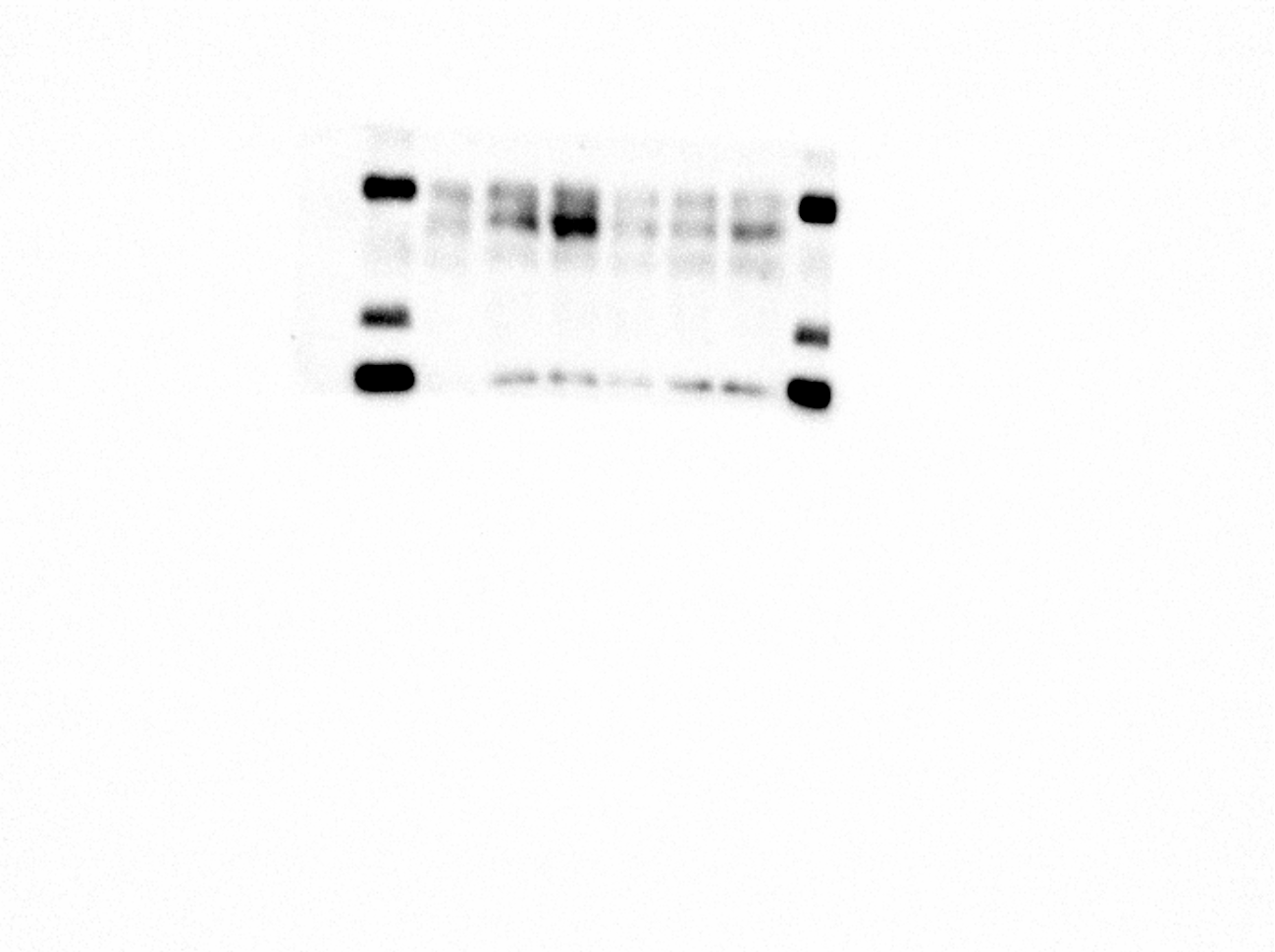

Supplement: Figure 4—source data 4. [file elife-94265-fig4-data4.zip › Figure 4-source data 4/Figure 4-source data 4_1.tif]

**D**

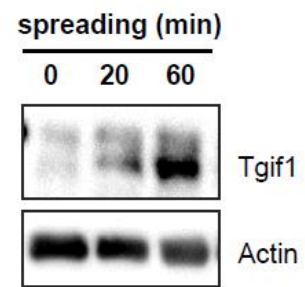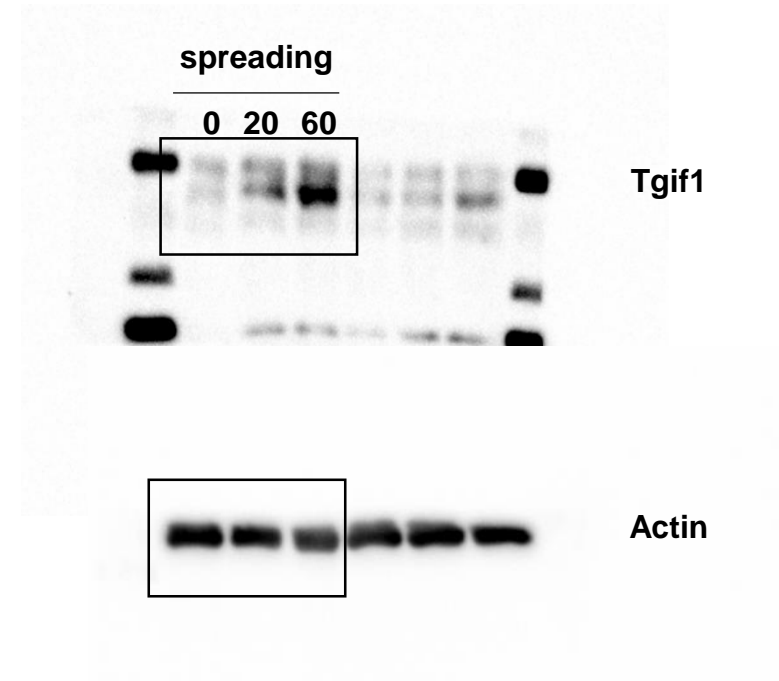

Figure 4D

Supplement: Figure 4—source data 5. [file elife-94265-fig4-data5.zip › Figure 4-source data 5/Figure 4-source data 5.pdf]

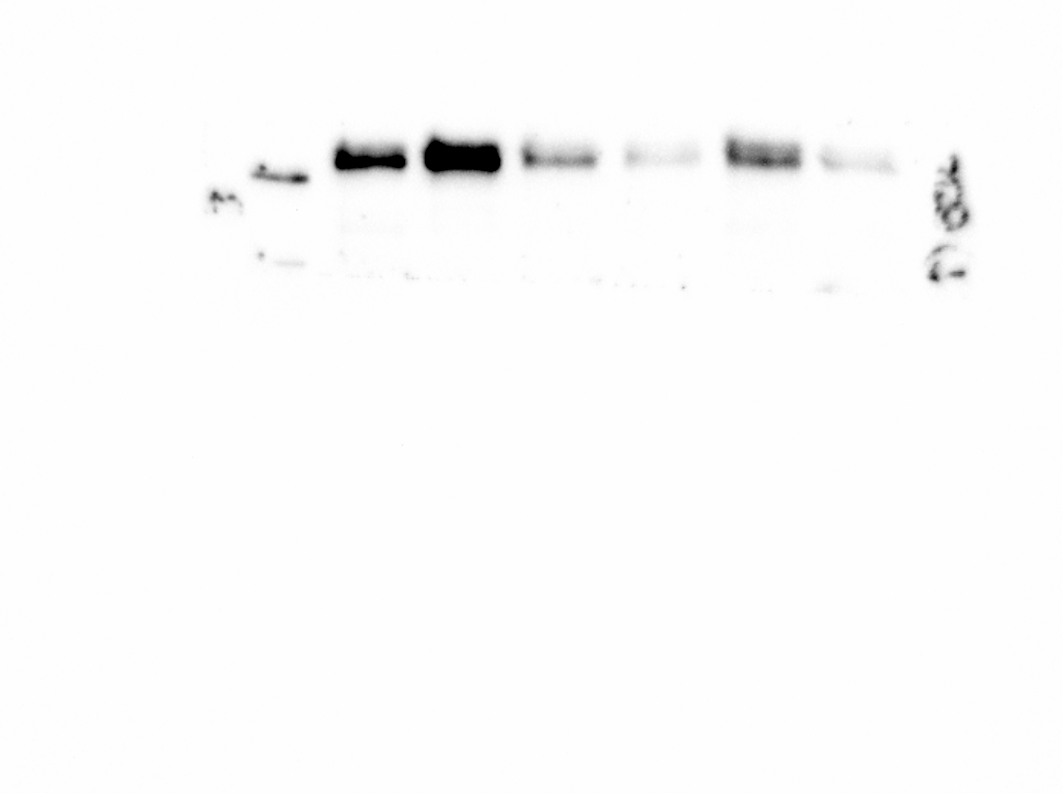

Supplement: Figure 4—source data 6. [file elife-94265-fig4-data6.zip › Figure 4-source data 6/Figure 4-source data 6_1.tif]

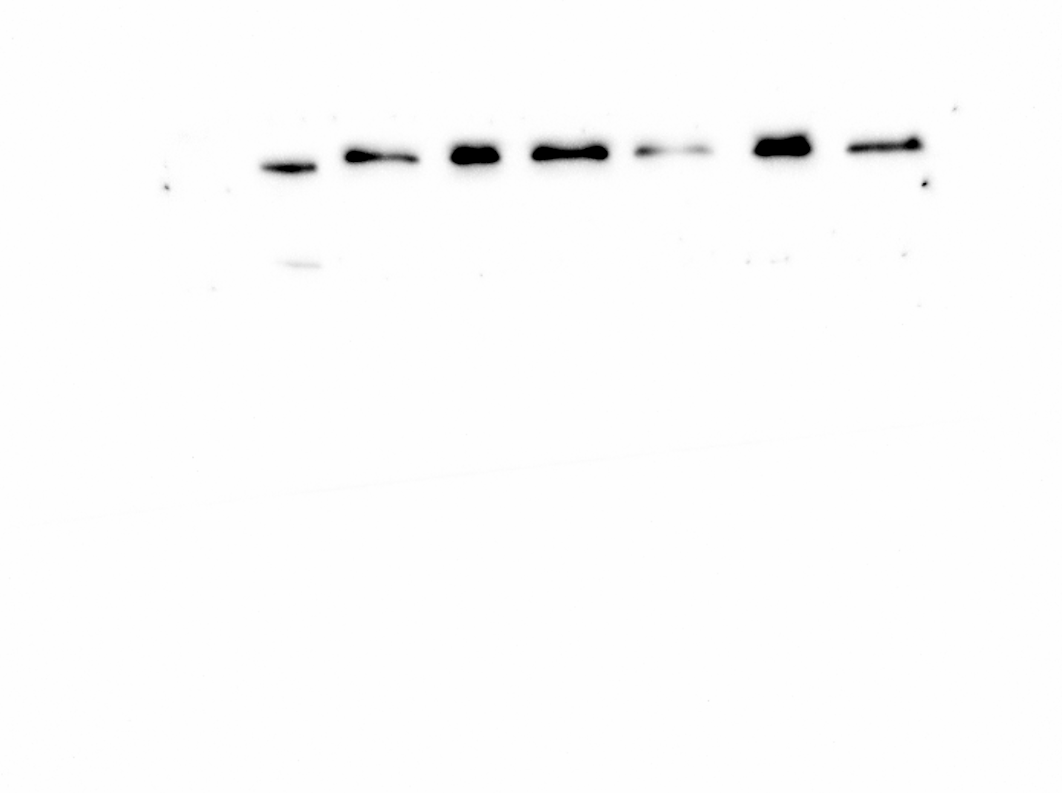

Supplement: Figure 4—source data 6. [file elife-94265-fig4-data6.zip › Figure 4-source data 6/Figure 4-source data 6_2.tif]

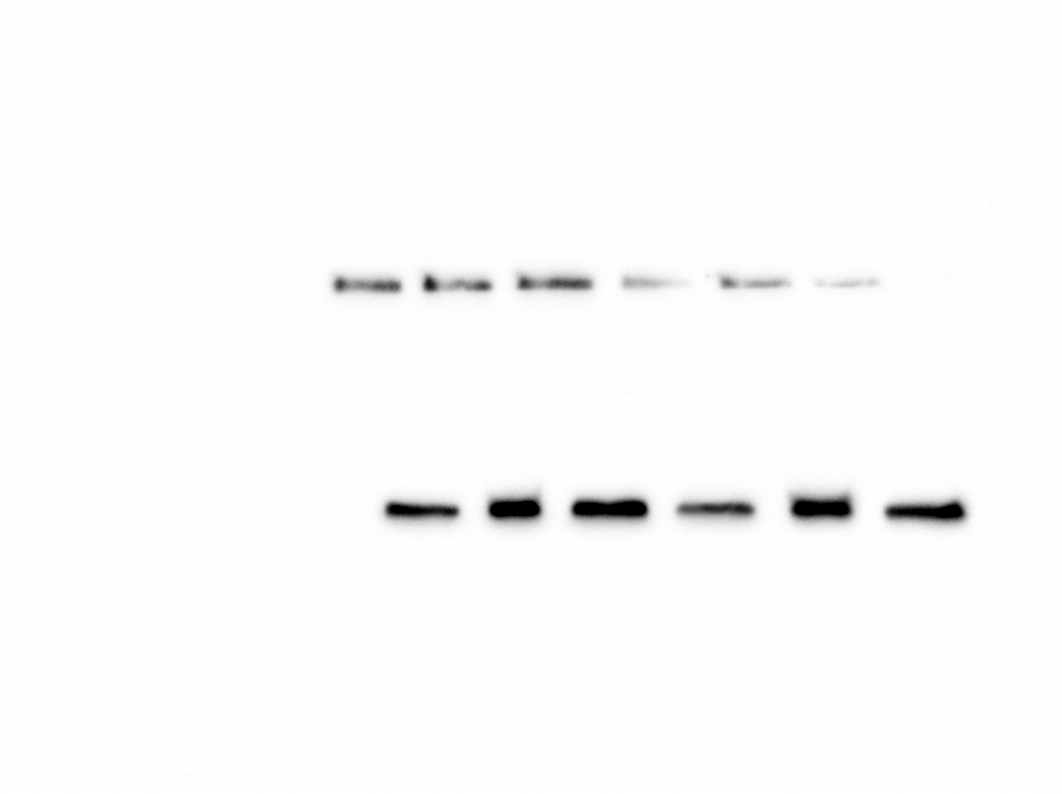

Supplement: Figure 4—source data 6. [file elife-94265-fig4-data6.zip › Figure 4-source data 6/Figure F- source data 6_3.tif]

**F**

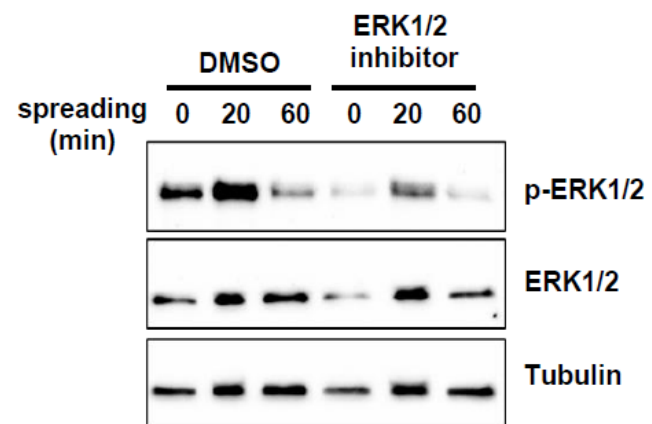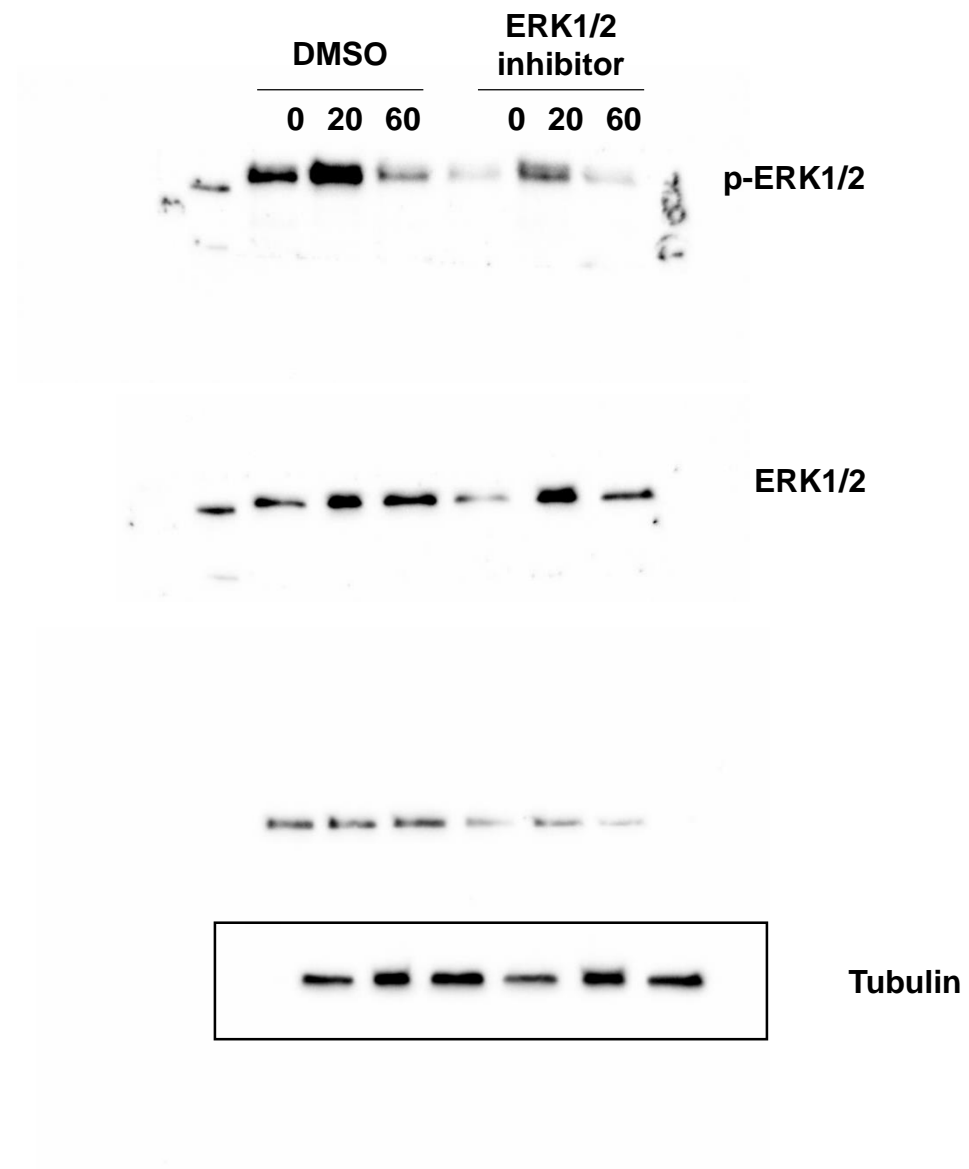

Figure 4F

Supplement: Figure 4—source data 7. [file elife-94265-fig4-data7.zip › Figure 4-source data 7/Figure 4F-source data 7.pdf]

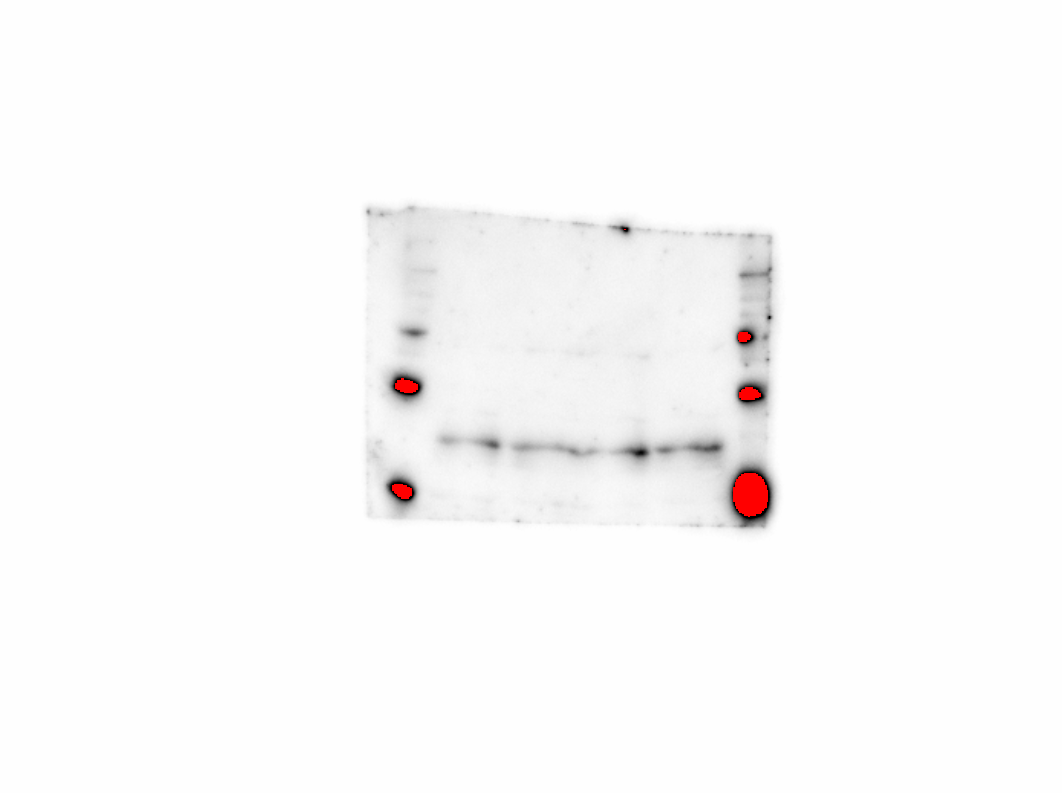

Supplement: Figure 7—source data 2. [file elife-94265-fig7-data2.zip › Figure 7-source data 2/Figure 7D-source data 2_1.tif]

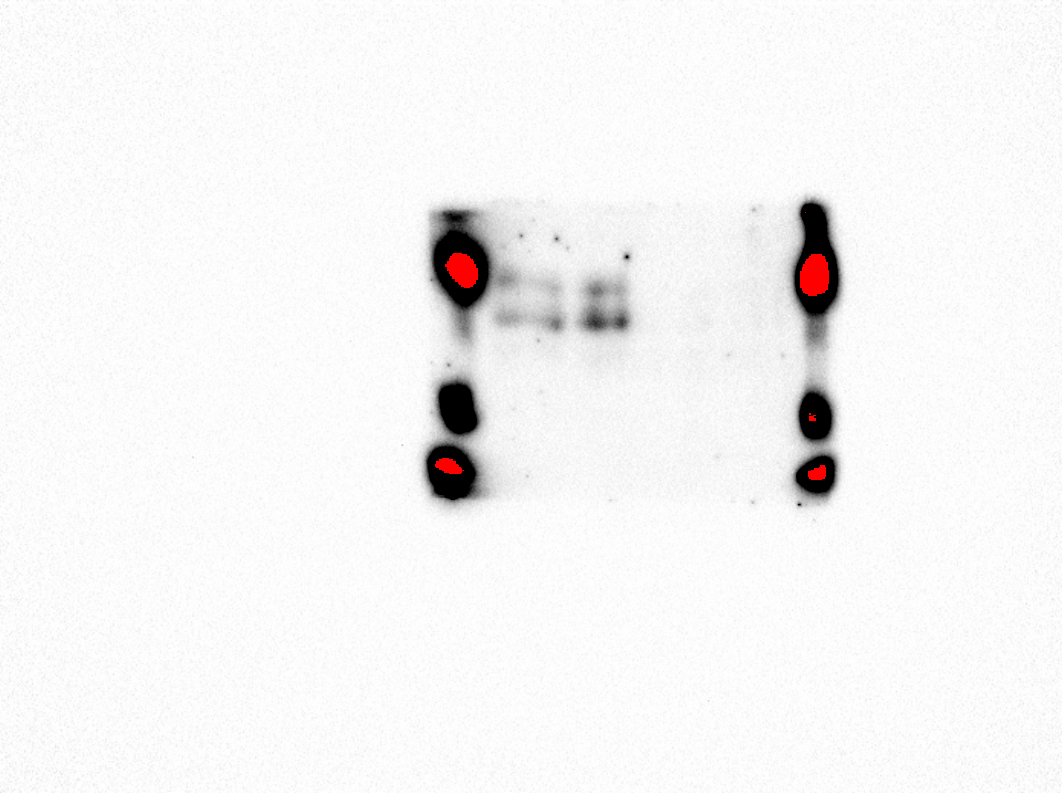

Supplement: Figure 7—source data 2. [file elife-94265-fig7-data2.zip › Figure 7-source data 2/Figure 7D-source data 2_2.tif]

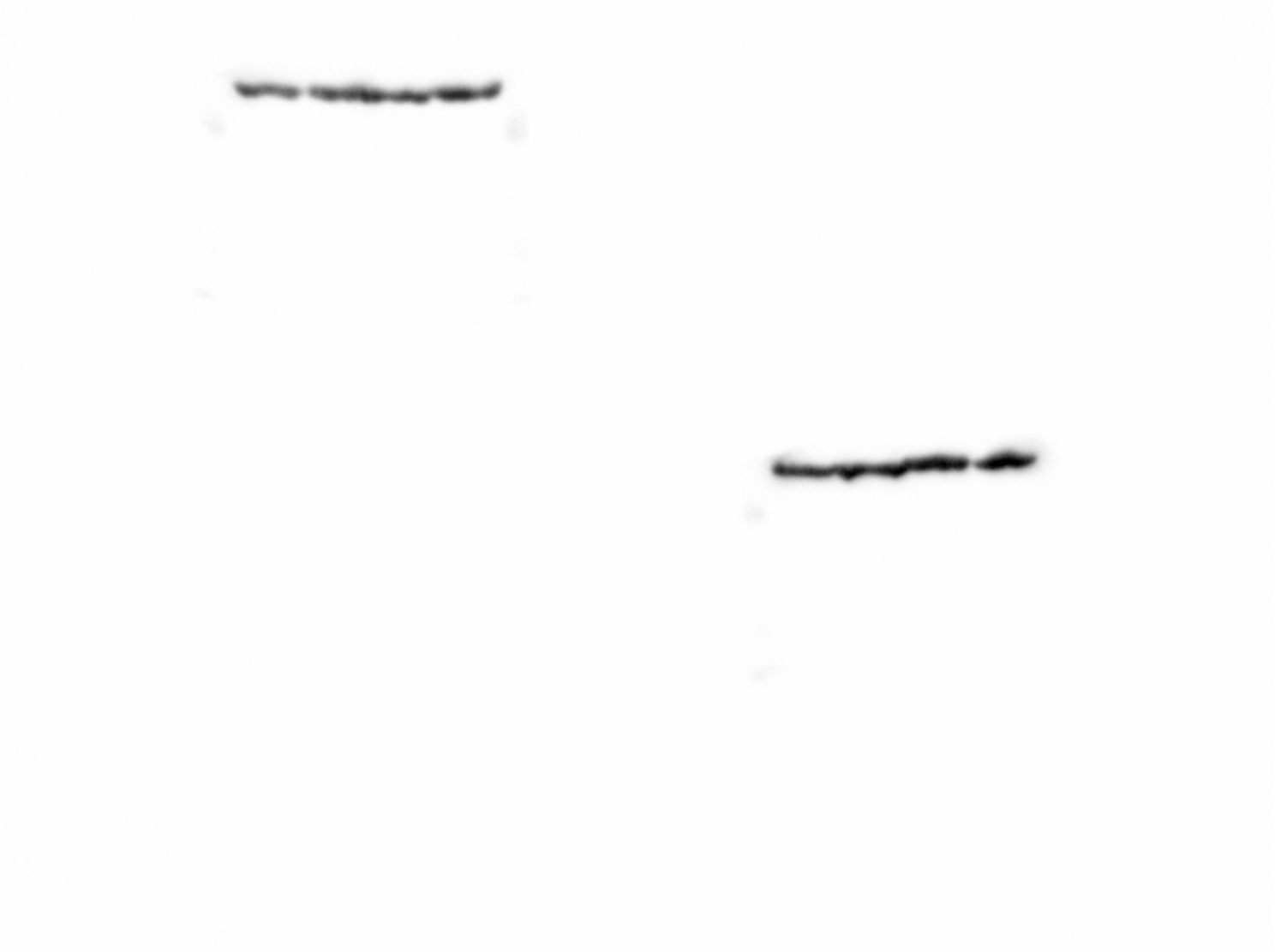

Supplement: Figure 7—source data 2. [file elife-94265-fig7-data2.zip › Figure 7-source data 2/Figure 7D-source data 2_3.tif]

**D**

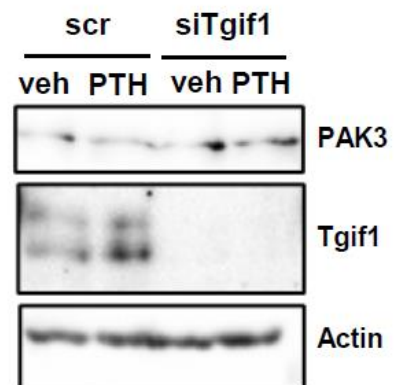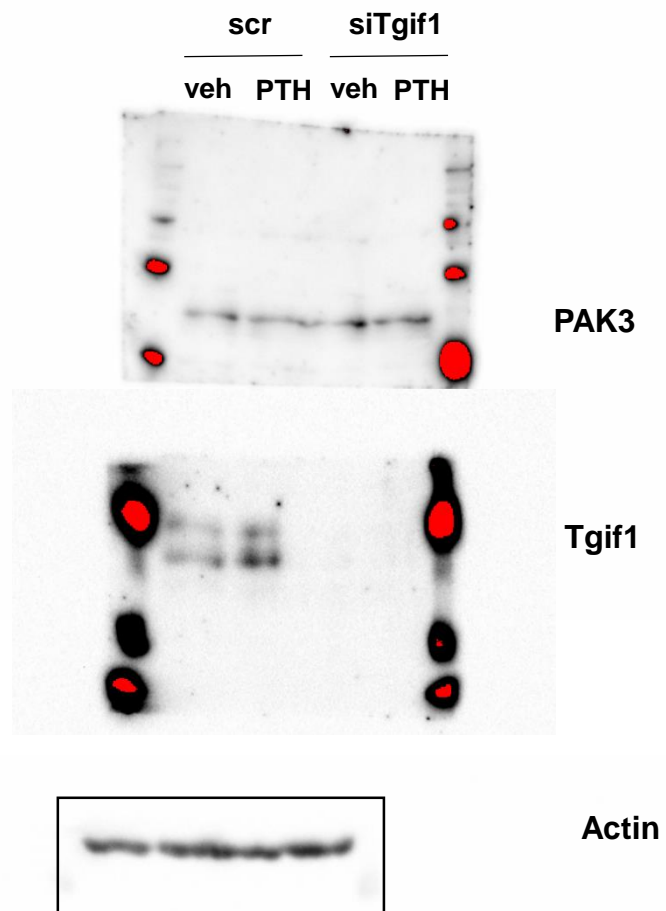

Figure 7D

Supplement: Figure 7—source data 3. [file elife-94265-fig7-data3.zip › Figure 7-source data 3/Figure 7D-source data 3.pdf]
